# An automated approach to the quantitation of vocalizations and vocal learning in the songbird

**DOI:** 10.1371/journal.pcbi.1006437

**Published:** 2018-08-31

**Authors:** David G. Mets, Michael S. Brainard

**Affiliations:** 1 Center for Integrative Neuroscience, University of California, San Francisco, San Francisco, California, United States of America; 2 Howard Hughes Medical Institute, University of California, San Francisco, San Francisco, California, United States of America; 3 Departments of Physiology and Psychiatry, University of California, San Francisco, San Francisco, California, United States of America; The Pennsylvania State University, UNITED STATES

## Abstract

Studies of learning mechanisms critically depend on the ability to accurately assess learning outcomes. This assessment can be impeded by the often complex, multidimensional nature of behavior. We present a novel, automated approach to evaluating imitative learning. Conceptually, our approach estimates how much of the content present in a reference behavior is absent from the learned behavior. We validate our approach through examination of songbird vocalizations, complex learned behaviors the study of which has provided many insights into sensory-motor learning in general and vocal learning in particular. Historically, learning has been holistically assessed by human inspection or through comparison of specific song features selected by experimenters (e.g. fundamental frequency, spectral entropy). In contrast, our approach uses statistical models to broadly capture the structure of each song, and then estimates the divergence between the two models. We show that our measure of song learning (the Kullback-Leibler divergence between two distributions corresponding to specific song data, or, Song *D*_KL_) is well correlated with human evaluation of song learning. We then expand the analysis beyond learning and show that Song *D*_KL_ also detects the typical song deterioration that occurs following deafening. Finally, we illustrate how this measure can be extended to quantify differences in other complex behaviors such as human speech and handwriting. This approach potentially provides a framework for assessing learning across a broad range of behaviors like song that can be described as a set of discrete and repeated motor actions.

## Author summary

Measuring learning outcomes is a critical objective of research into the mechanisms that support learning. Demonstration that a given manipulation results in better or worse learning outcomes requires an accurate and consistent measurement of learning quality. However, many behaviors (e.g. speaking, walking, and writing) are complex and multidimensional, confounding the assessment of learning. One behavior subject to such confounds, vocal learning in Estrildid finches, has emerged as a vital model for sensory motor learning broadly and human speech learning in particular. Here, we demonstrate a new approach to the assessment of learning for complex high dimensional behaviors. Conceptually, we estimate the amount of content present in a reference behavior that is absent in the resultant learned behavior. We show that this measure provides a holistic and automated assessment of vocal learning in Estrildid finches that is consistent with human assessment. We then illustrate how this measure can be used to quantify changes in other complex behaviors such as human speech. We conclude that this approach could be useful in assessing shared content in other similarly structured behaviors composed of a set of discrete and repeated motor actions.

This is a *PLOS Computational Biology* Methods paper.

## Introduction

**Fig 1:**
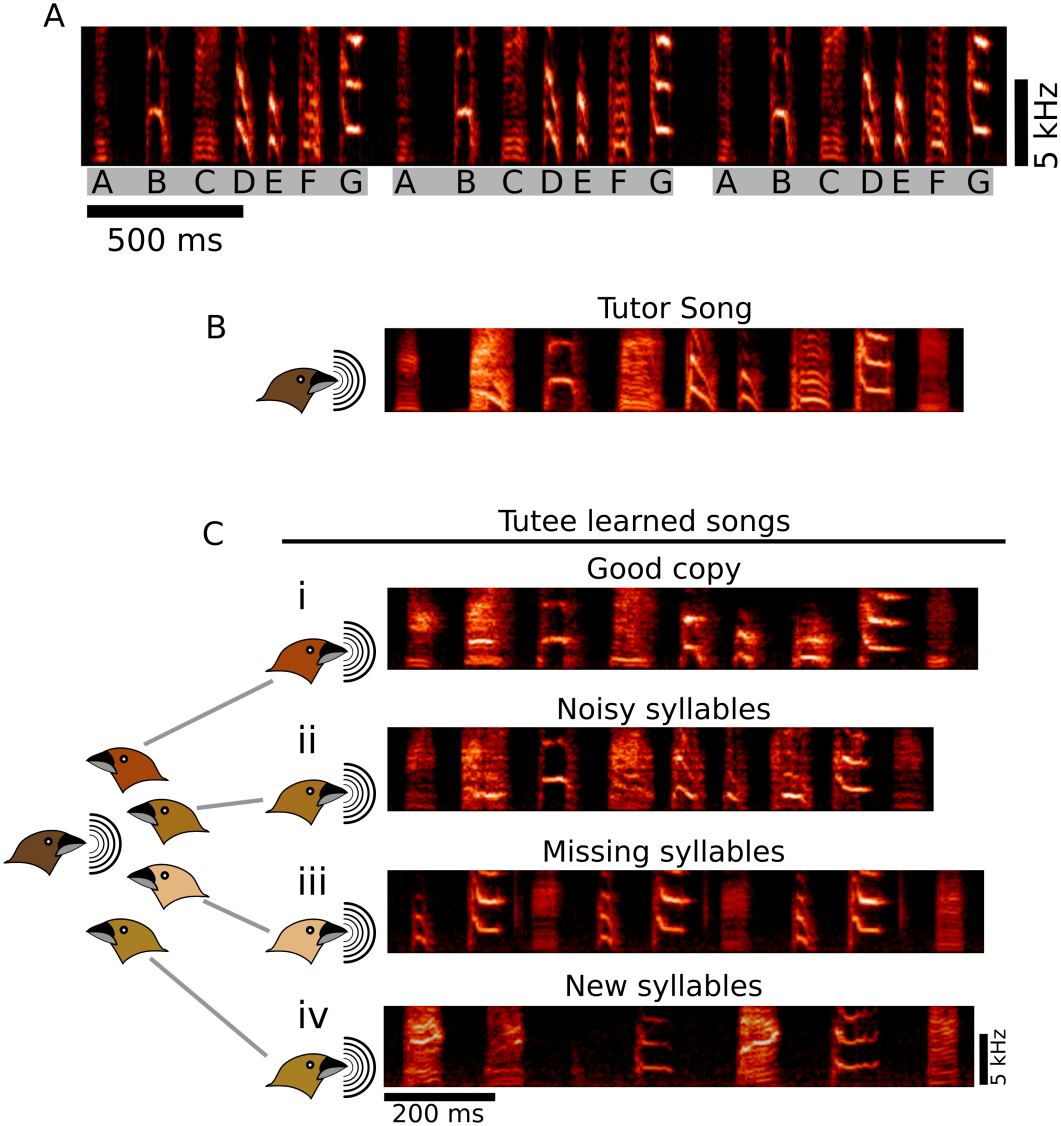
Quantification of song learning is complicated by variety in both learning and failure to learn. (A) Typical sample of song from an adult Bengalese finch. Song is composed of a set of categorically distinct syllable types (labeled ‘A’, ‘B’, ‘C’…) that are organized into larger, repeated, sequences (gray bars). Both the spectral structure of syllables and their sequencing are learned features of song. Hence, song is a complex, high dimensional behavior that differs across individuals. (B) Song of an adult male ‘tutor’ and (C) songs of four juvenile ‘tutees’ that were all exposed to the same tutor song, illustrating variation in the quality of song learning. (Ci) Song from a tutee that learned the spectral content of the tutor song well, producing a song with accurate copies of all syllables. (Cii) Song from a tutee that copied all syllables, but with noisier versions than those present in the tutor song. (Ciii) song from a tutee that failed to copy some of the syllables from the tutor song. (Civ) song from a tutee that included ‘new syllables’ that were not clearly present in the tutor song.

Songbird vocal learning shares many parallels with speech learning and is a powerful and tractable model system for elucidating neural and behavioral mechanisms underlying vocal control and vocal learning [1,2]. Birds, like humans, learn vocalizations early in life through exposure to the vocalizations of an adult ‘tutor’ followed by a period of practice that eventually results in typical adult vocalizations that require auditory feedback for maintenance [1]. Song is composed of discrete units of sound (syllables) organized into higher order sequences [3]. In the finch species examined here, a given bird’s song comprises about 5–10 categorically distinct syllable types, with these distinct types defined by their unique spectro-temporal structure (Fig 1A). Hence, an individual bird’s song can be described as a specific set of categorically distinct syllable types (that can be labeled ‘A’, ‘B’, ‘C’ and so on).

Qualitatively, learning (and failure to learn) can occur in different ways (Fig 1B and 1C) [4]. For example, juvenile ‘tutees’ could learn to produce all distinct syllable types present in an adult ‘tutor’ song, but the spectral content of the syllables might be imperfect or noisy (Fig 1Ci-1Cii), while other tutees might completely fail to learn some syllables (Fig 1Ciii), and still others might improvise new syllables (Fig 1Civ).

Because of these complexities, many studies have relied on human evaluation of song similarity and learning [5–8]. Indeed, human scorers can provide a useful ‘holistic’ assessment of similarity between complex behaviors, such as songs, which integrates across many stimulus dimensions. However, human scoring suffers from several problems including 1) it requires scorers to be trained on species-specific vocalizations, and analysis of different vocalization types often requires new training, 2) correspondingly, scores are potentially inconsistent over time and across different evaluators, and 3) human scoring is labor intensive and does not readily scale to the size of relevant datasets, which, in the case of birdsong, can include many individuals and thousands of vocalizations per day.

More recent attempts at quantification of song similarity have focused on assessing learning based on specific, reliably and automatically measured features of song (e.g. the fundamental frequency of a specific syllable, or song entropy) [9–12]. In these approaches, selected samples of songs are decomposed into sets of feature values and song similarity is then evaluated as the similarity between weighted feature sets associated with those samples, overcoming some of the inter-evaluator variability associated with human scoring, and additionally enabling useful assessment of how specifically analyzed features such as the ‘pitch’ or ‘noisiness’ of syllables differ across songs and conditions [13]. However, these approaches can still require significant human intervention for the selection of which samples of song material to analyze and which features to weigh in assessing similarity.

Here, we present an approach to scoring the similarity between pairs of songs (and other complex stimuli) that reproduces some of the human capacity for holistically integrating across complex stimulus dimensions but that is also automatic, reproducible and efficiently deployed across large data sets. Our measure is intended to quantify the amount of spectral content that is present in a reference song (e.g. the tutor song), but absent from a comparison song (e.g. the song of a tutee). To accomplish this, we first transform each song into a distribution of features in a ‘similarity-space’. We then quantify the difference between songs as the Kullback-Leibler divergence (*D*_KL_) between the corresponding distributions. We validate our approach by measuring the *D*_KL_ between pairs of songs for two conditions. First, we assess song learning by comparing adult tutor songs and juvenile tutee songs in the Bengalese finch (*Lonchura striata domestica*), a species with variability in both spectral content and syntax. We show that the *D*_KL_ provides a measure of the quality of song learning in Bengalese finches that is well correlated with scores provided by human experts. Second, we assess song deterioration following deafening by comparing baseline songs produced by adult zebra finches (*Taeniopygia guttata guttata*) with songs of the same individuals at varying times following deafening. We show that *D*_KL_ detects and quantifies the characteristic song deterioration that follows deafening [7,14]. Finally, we illustrate how this approach can be extended to quantify similarity for other complex behaviors such as human speech.

## Results

### Transformation of song into syllable similarity-space

Our comparison of songs is based on the set of discrete vocalizations (syllables) produced by each bird. We treat each syllable as a high dimensional ‘feature’ and each song is then represented by the distribution of features associated with the constituent syllables. Our measure of song similarity is intended to quantify how similar are the distributions of these features across songs. Hence, a central aspect of our approach is to transform the features of each song into a parameter space where the distribution of features of one song can be meaningfully compared with the distribution of features of a second song. Desirable characteristics of this space are 1) that it is rich enough to capture relevant variation in the behavior, and 2) that it structures the behavioral data such that points that are closer together in the space are more similar to each other by human judgment. We describe below how we transform songs into a syllable ‘similarity-space’ intended to achieve these characteristics and then provide examples that illustrate how this transformation empirically restructures song data in a way that facilitates subsequent assessment of similarity between songs.

The essence of the transformation of song data into similarity-space is that it represents each syllable based on its similarity to a large number of other syllables that are representative of the bird’s repertoire. In the examples that follow, we represent each syllable by its similarity to each of 50 ‘basis syllables’ that are randomly drawn from the bird’s repertoire (we later examine the effects of varying the number of basis syllables). Hence, each syllable is represented by a feature vector of length 50, where each element of the vector quantifies similarity to one of the basis syllables. This contrasts with an approach in which each syllable is represented by a feature vector that is composed of specific user defined measurements, such as pitch, amplitude, entropy, duration, etc. [10–12,15,16]. We use such a vector of syllable similarities to represent each syllable based on two related ideas. First, we supposed that the dimensions of this space would be well matched to the natural range of variation of syllables (because each dimension of the space by definition represents similarity to a randomly selected instance of a syllable). This is analogous to the idea that it might be effective to represent an item of fruit, such as a red Fuji apple, as a vector representing the similarity of that specific fruit to a large number of instances of other fruits (including other apples, as well as oranges, bananas, watermelon, etc.). Second, we supposed that more similar syllables might be clustered closer together in this space, with members of a given type (i.e. different variants of syllable A) in closest proximity, and members of structurally similar types in closer proximity than members of more distinct types—analogous to the idea that in ‘fruit space’ multiple instances of Fuji apples would be clustered closest together, with other types of red apples nearby, and green apples, oranges and watermelon progressively further away. The transformation into similarity-space also reduces the dimensionality of the representation of each syllable to 50, from a value of 3200 for the corresponding raw waveform (for a 100ms sampled at 32kHz).

Transformation into similarity-space requires a choice of how to quantify the similarity between each sample syllable and the basis syllables. We examined a variety of approaches to measuring similarity between syllables and found empirically that the ultimate assessment of song learning was quite robust across these measures. An intuition about why this might be the case is that our method relies on the distribution of syllables in similarity-space and that, regardless of the similarity metric, two acoustically similar samples of a given syllable will have feature vectors that are close to each other, and hence those syllables will be projected into nearby locations in similarity-space. In the main results that follow, we use the Euclidean distance between the power spectral densities (PSDs) of pairs of syllables as a metric for similarity (see Methods). This is a simple and computationally efficient metric that explicitly ignores time varying spectral structure, but that nevertheless proves sufficient to capture relevant differences in song structure that correlate with human judgments. We later consider how other syllable representations, including one that captures time varying spectral structure influences performance.

**Fig 2:**
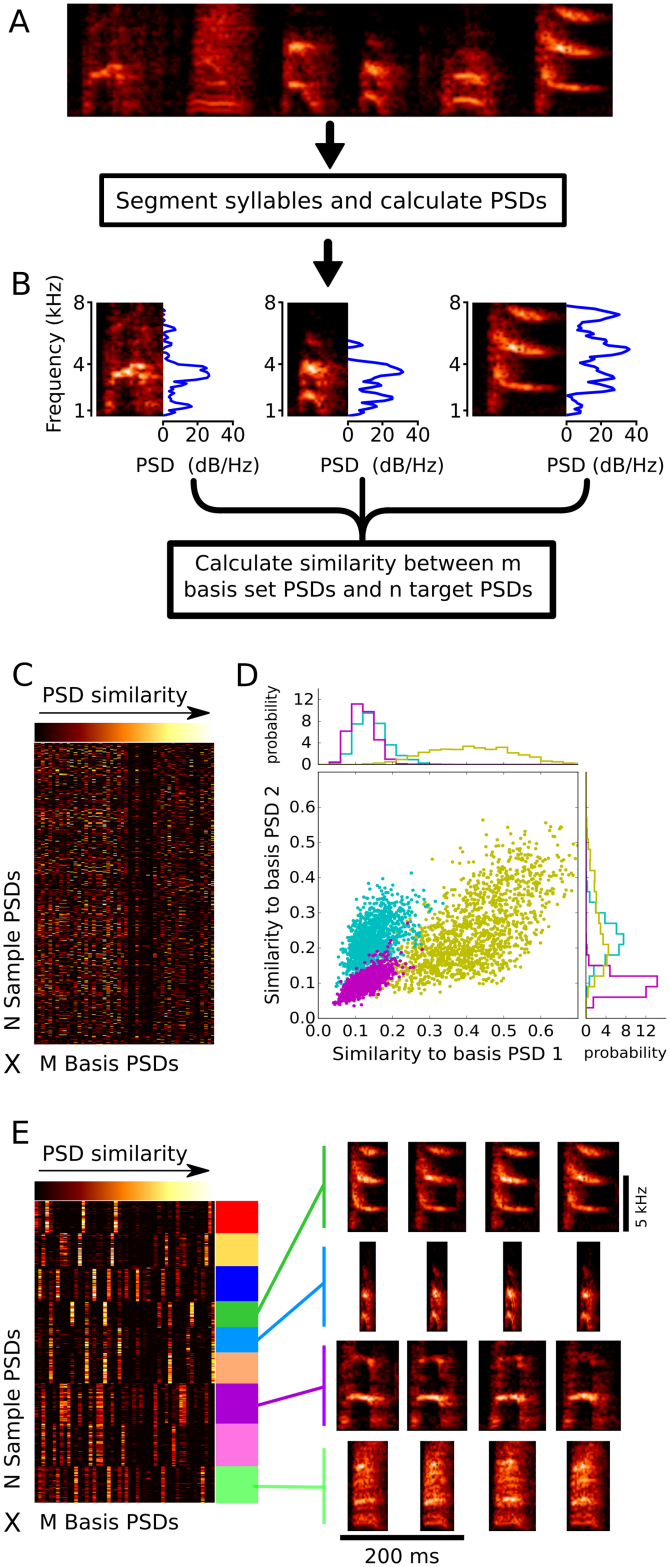
Transformation of song data into syllable similarity-space. (A) To transform data from a given bird into similarity-space we first segment all syllables from a set of songs produced by that bird and compute their corresponding PSDs. (B) Three examples of segmented syllables, each of a different type, and their corresponding PSDs. (C) For each of 3000 sample syllables from the song to be analyzed, similarity of PSDs is calculated relative to a basis set of PSDs for 50 syllables randomly drawn from the same song. This creates an M (number of basis syllables) by N (number of sample syllables) similarity matrix. (D) Visualization of how transformation of raw syllable data into the syllable similarity-space results in a clustering of syllables by type. Each point in the plot indicates the similarity between the PSD for one sample syllable and two basis PSDs (‘basis PSD1’ and ‘basis PSD2’) from the set of 50 basis PSDs. For clarity of exposition, only data that fall into one of three regions of high density are plotted here. Each of these regions corresponds approximately to multiple instances of one syllable type (which cluster near each other because of the similarity in their PSDs). In practice, there were more than three regions of syllable clustering (corresponding approximately to the number distinct syllable types in the bird’s song), and these regions were represented in the 50 dimensional space defined by the basis set of PSDs (only two of which are illustrated here). The regions of high density in this similarity-space were fit with a Gaussian mixture model, in which the optimal number of Gaussian mixtures was determined by Bayesian Information Criteria. Individual data points here are color-coded by their assignment to one of three Gaussian mixtures. For clarity of presentation, data from only three of the 9 total Gaussian mixtures are shown. In any single dimension (top and right) data points assigned to each Gaussian mixture were approximately normally distributed. (E) Similarity matrix shown in C, reordered so that data are grouped by assignment to each of 9 Gaussian mixtures fit to the data (represented by colored blocks at the right of the similarity matrix). In this reordered representation, it is apparent that syllables assigned to each Gaussian mixture have a shared ‘bar code’ reflecting a shared pattern of PSD similarity values relative to the basis PSDs. The spectrograms at the right illustrate that syllables assigned to a given Gaussian mixture tend to be of the same type.

An example of transformation of song into similarity-space is schematized in Fig 2. Starting with song data from a given bird (Fig 2A), we identify individual syllables as continuous amplitude traces above a threshold. For each syllable, we calculate the PSD using Welch's method (Fig 2B) [17]. We then calculate the Euclidean distance between each PSD and a “basis set” of PSDs (see Methods), where the basis set is a random draw from the set of PSDs for all syllables in the song. These computed similarities create an NxM matrix (Fig 2C) where N is the number of PSDs being analyzed ('sample' PSDs) and M is the number of PSDs in the basis set ('basis' PSDs). For the analyses presented below, we use a value for ‘N’ of 3000 PSDs drawn from the song to be modeled, and a value for ‘M’ of 50 PSDs to form the basis set for construction of the syllable similarity matrix.

Transforming the raw syllable data into syllable similarity-space has the important consequence that it naturally and automatically clusters syllables with similar PSDs into a small number of regions of high density. Each of these regions corresponds approximately to a group of syllables that would be identified by a human observer as belonging to a specific type. That is because each sample of a syllable type has a similar PSD, and therefore each of these samples will have a similar pattern of distances from each of the elements of the basis set. This is illustrated in Fig 2D, which plots the similarities between sample PSDs for three discrete syllable types (blue, purple and yellow points) and two basis PSDs (out of a total of 50 basis PSDs). There is a broad dispersion of points corresponding to each syllable type, which reflects variation in the acoustic structure across multiple renditions of each syllable type. However, even in just two dimensions, the clusters of points corresponding to each syllable type are well separated. Importantly, this separation depends on the pattern of similarities across both of the basis PSDs considered jointly; the blue and purple samples are largely overlapping in their similarities to basis PSD 1 (as indicated by the marginal distributions plotted at the top), while the blue and yellow points are largely overlapping in their similarities to basis PSD 2 (as indicated by the marginal distributions plotted at the right). Similarly, across all 50 basis PSDs, samples of each of the 9 syllable types from this bird’s song were clustered into distinct groups. This is illustrated in Fig 2E, which depicts for each of 3000 sample PSDs the feature vector of similarities to the 50 basis PSDs. These are the same data as in Fig 2C, but have been reordered to group together PSDs that have similar feature vectors. Each group (indicated by colored blocks at the right) is characterized by a stereotyped pattern of similarities across the 50 dimensions of the basis set (these ‘bar codes’ are apparent in the color scale representation of PSD similarity in Fig 2E). These groups that are defined by proximity in similarity-space generally correspond to syllables of a given type, as illustrated by the example spectrograms plotted at the right for 4 of the groups. Hence, the transformation into similarity-space results in a representation of song in which thousands of sample syllables are clustered into a small number of high density regions, corresponding approximately to the numbers and identities of categorically distinct syllable types in the original song.

**Fig 3:**
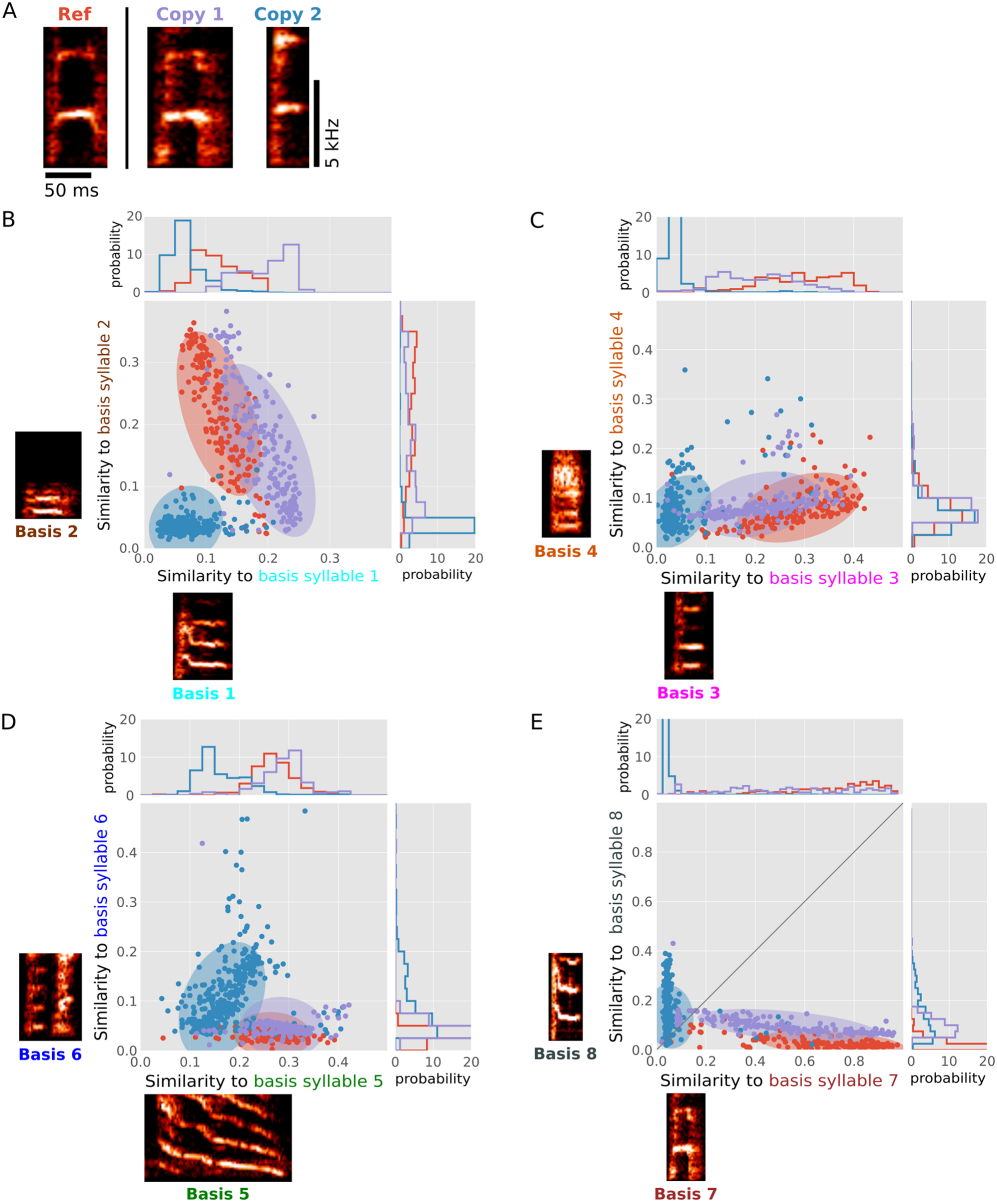
Syllables with similar spectral structure have overlapping distributions in syllable similarity-space. For this analysis, for each of three birds, instances of a specific syllable type, corresponding to ‘harmonic stacks’ were identified by human inspection. (A) Spectrograms of exemplar syllables produced by each of three birds (Ref, Copy 1, Copy 2). (B-E) Distributions corresponding to syllables of a specific type (exemplars in panel A) produced by three birds (Ref (red), Copy 1 (purple), Copy 2 (blue)) in similarity-space. Consistent with the human perception that Copy 1 and Ref are more similar to each other than either is to Copy 2, in all 4 panels, distributions produced by Ref (red) and Copy 1 (purple) are more overlapping than distributions produced by Copy 2 (blue) and either Ref or Copy 1. For all panels, the marginal distributions in each single dimension are depicted above and to the right and the basis syllables are depicted above and below. Ellipses are 80% confidence intervals (1.28 standard error) derived from a multivariate Gaussian fit to each set of syllable similarities. Throughout, colors indicate bird identity.

An additional characteristic of the transformation of PSDs into similarity-space is that it tends to place PSDs corresponding to more similar syllables in closer proximity than more dissimilar syllables. An example of this is illustrated in Fig 3, which shows the distribution in similarity-space of samples of three different syllable types (with examples shown as spectrograms in Fig 3A). They include a ‘harmonic stack’ syllable from a reference song (‘ref’), and copies of the same syllable that were learned by two different birds. One bird produced a copy that was subjectively a good match to the reference syllable (‘Copy 1’), while the second bird produced a noticeably worse copy (‘Copy 2’). Each of the panels in Fig 3B–3E shows distributions of samples of the three syllables in two dimensions of syllable similarity-space (with each dimension defined by similarity to a different basis PSD). The distributions of points corresponding to each syllable were broadly dispersed and overlapping in any given dimension. However, consistent with subjective impressions of syllable similarity, the distributions corresponding to renditions of the reference syllable (red points) was generally more overlapping with the distributions corresponding to the better copy (Copy 1, purple points) than the distributions corresponding to the worse copy (Copy 2, blue points).

### Quantification of differences between reference and comparison song models

The examples above (Fig 3) illustrate the motivation for our metric of song similarity, which quantifies the degree of overlap in similarity-space between the distributions corresponding to syllables from a reference song and comparison song. Unlike the examples in Fig 3, this overlap is computed across a large sample of all syllables from each song rather than just samples of a single user-identified syllable. However, when two songs each contain acoustically similar copies of the same syllable, the samples corresponding to that syllable will occupy overlapping regions of similarity-space. Because we are interested in measuring the degree to which a reference song (such as a tutor song) is copied by a comparison song (such as the song of juvenile ‘tutee’ that learned from the tutor), our goal is to quantify how much of the distribution of syllables corresponding to the reference song is accounted for by the distribution corresponding to the comparison song. To do so, we first model the distributions that correspond to samples of the reference and comparison songs. We then quantify how well the distribution corresponding to the comparison song matches the distribution corresponding to the reference song.

In order to model the distributions of points in similarity-space that correspond to samples of a given song, we fit the sample points with Gaussian Mixture Models (GMMs). We use GMMs, in part because the marginal distributions corresponding to individual syllable types have qualitatively Gaussian shapes. This can be seen by examining the marginal distributions for the example syllables plotted in Figs 2D and 3B–3E (plotted outside the axes corresponding to each dimension in similarity-space). We fit a series of GMMs with increasing numbers of mixture components to the sample data using expectation maximization [18,19], and the model with the best fit to the data based on the lowest Bayesian Information Criterion [20] is then used to represent the song of that bird. Because the data corresponding to different syllables types tends to fall into discrete regions of high density in similarity-space (e.g. yellow, purple and blue points in Fig 2D), the number of Gaussian mixture components in each song model corresponds approximately to the number of discrete syllable types present in the song.

Because we capture the spectral content of each song as a statistical model, we can compare the spectral content of the reference and comparison songs using a principled information theoretic measure. Specifically, we estimate the Kullback-Leibler divergence (*D*_KL_) between those distributions (see Methods) [21]. In this context, the *D*_KL_ captures content contained in the reference song that is absent from the comparison song. Hence, a larger *D*_KL_ corresponds to worse learning (greater divergence between the song distributions). We refer to this measure as the Song *D*_KL_.

**Fig 4:**
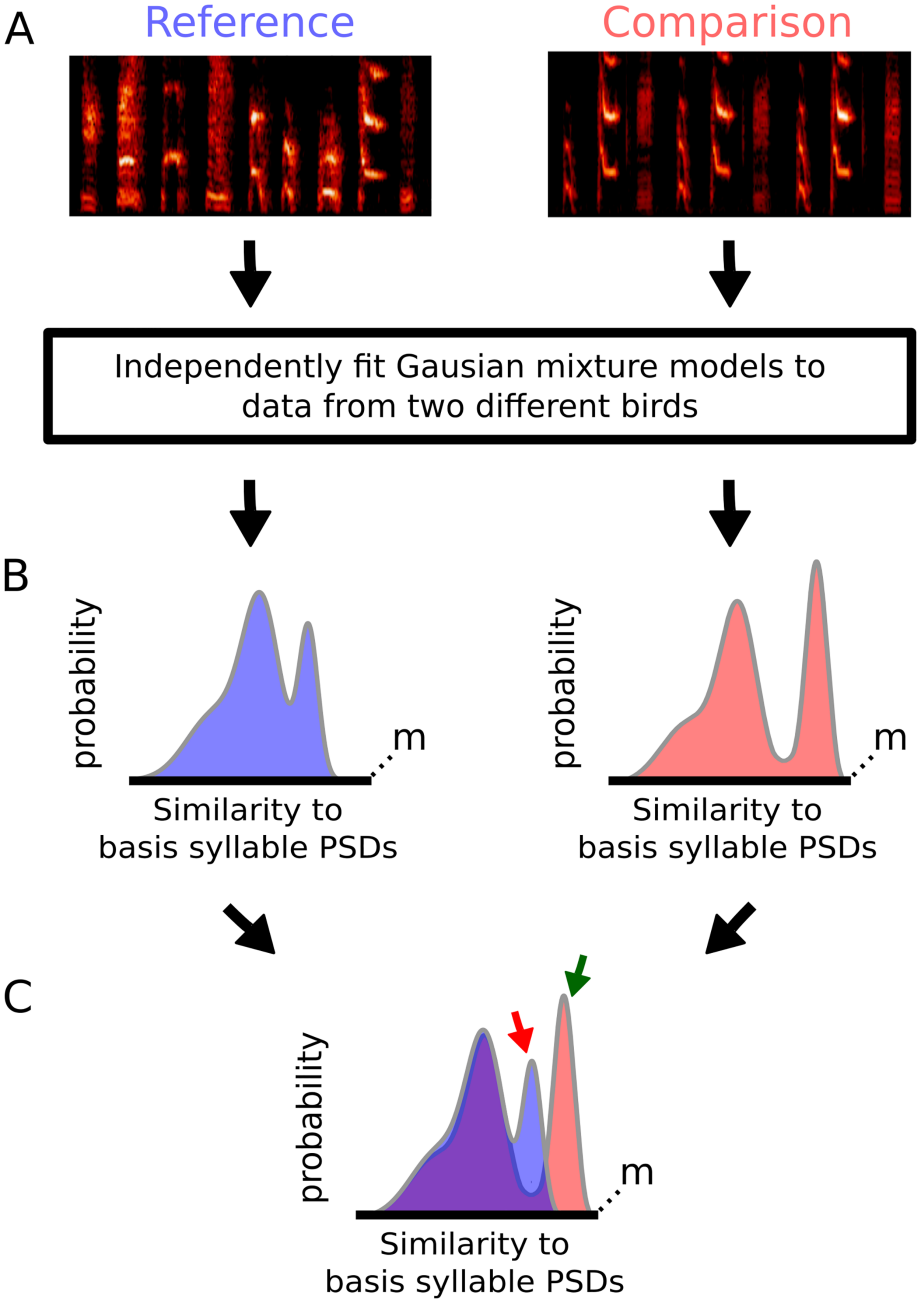
Estimation of the amount of spectral content present in the reference (tutor) song that is absent from the comparison (tutee) song. (A) Example reference and comparison songs. To compute the *D*_KL_ for these songs, we first fit Gaussian mixture models (GMMs) to the data from each song. (B) Representation in one dimension of the GMMs fit to song spectral content for both the reference song (left, blue) and the comparison song (right, red). (C) Superimposed mixture models for the reference song (blue) and comparison song (red). Regions of the reference-song mixture model which are not shared with the comparison-song model (red arrow) correspond to reference song content which is absent in the comparison song and will result in a higher *D*_KL_. However, regions of the comparison-song model which are not shared with the reference-song model (green arrow) will not impact the *D*_KL_.

An illustration of how the *D*_KL_ quantifies differences between two songs is provided in Fig 4. Syllables from a reference song and comparison song are transformed into the same syllable similarity-space (defined by a basis set of PSDs drawn randomly from the reference song). The distributions of syllables corresponding to each song are then fit by GMMs, represented here in one dimension (Fig 4B and 4C). These GMMs are superimposed in Fig 4C. In this case, it is apparent that there is significant overlap between the reference and comparison models, reflecting generally good match between the songs. However, there are some portions of the reference model that are poorly fit by the comparison model (e.g. Fig 4C, red arrow). These correspond to regions in similarity-space that are occupied by samples from the reference song without corresponding samples from the comparison song. Such differences could reflect syllables in the reference song that were either poorly copied or completely omitted in the comparison song. The *D*_KL_ is estimated as the log-likelihood of samples drawn from the reference distribution minus the log-likelihood of the same (reference) samples under the comparison distribution. Hence, regions such as this (where samples are likely under the reference distribution but unlikely under the comparison distribution, red arrow) result in larger values of Song *D*_KL_, corresponding with worse learning. In contrast, because the *D*_KL_ is asymmetric (depending only on the likelihood of points drawn from the reference distribution), it is not affected by portions of the comparison model that are poorly fit by the reference model (e.g. Fig 4C, green arrow). Hence, *D*_KL_ will be low if the tutee (comparison) learned all elements of the tutor (reference) song well, but will be unaffected by tutee song content that is not present in the tutor song (e.g. song content innovated by the tutee; we discuss later how our approach can be extended to quantify innovation by the tutee).

A critical aspect of our similarity measure is that it quantifies similarities in the distributions corresponding to a large number of samples from each song. This contrasts with an approach of identifying a small number of exemplar songs or representative syllables and measuring the differences in their mean structure. The consequences of this difference are illustrated by the example syllables plotted in Fig 3. In this case, individual samples of each of the represented syllables are broadly dispersed. This broad dispersion of points reflects variation across the individual renditions of a given syllable type, and that those different renditions can have widely varying Euclidean distances to a given basis syllable. For instance, ‘basis syllable 7’ in Fig 3E is a specific instance of the ‘harmonic stack’ reference syllable. However, while some samples of the reference syllable (red points) have a high similarity to basis syllable 7 (values close to 1.0), others have very low similarity (values approaching 0). Here, this variation in similarity to basis syllable 7 results in part because small differences in the fundamental frequency of two ‘harmonic stack’ syllables can result in PSDs in which the peaks in the spectra are misaligned (and Euclidean distances are large). A measure of similarity that simply compared individual exemplar syllables (or the means of two syllable types) from the reference song and another song would be subject to large nonlinearities of this sort (given a simple Euclidean metric). However, our measure, which compares the shapes of the distributions corresponding to those syllable types across many renditions and multiple dimensions, effectively captures varying degrees of overlap in syllable structure despite the broad dispersion of individual samples (Fig 3B–3E).

### Song *D*_KL_ closely parallels human assessment of learning outcomes

**Fig 5:**
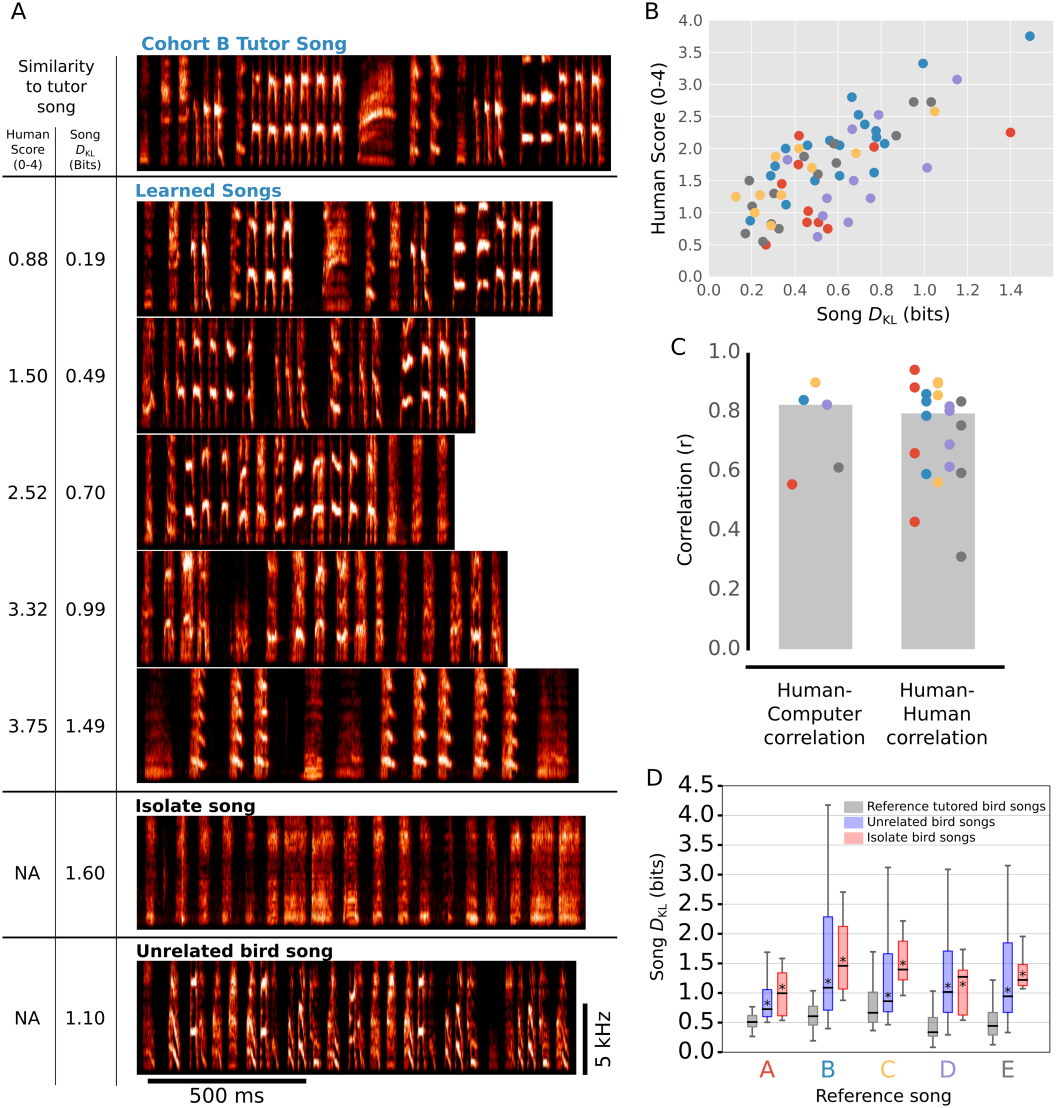
Song *D*_KL_ closely parallels human assessment of learning outcomes. The quality of learning for individuals from five cohorts, each with a distinct tutor song, were evaluated by song *D*_KL_ and human inspection. (A) Example spectrograms of the tutor song from one cohort and the songs of 5 tutees from the same cohort (cohort B). Also shown, for comparison, is the song of one isolate bird raised without tutor song exposure (isolate song) and the song from one bird raised with a different tutor (unrelated bird song). Numbers at left indicate the *D*_KL_ and human similarity scores for each song relative to the tutor song from cohort B. (B) There was a good correspondence between song *D*_KL_ and human evaluations of learning across a broad range of song similarities. Here, human scores are the average of four human judges. Across all five cohorts, *D*_KL_ and human scores were well correlated (p < 0.01, r = 0.722, OLS). (C) Comparison of song *D*_KL_ and human scores for each of the five cohorts. Human-computer correlation (left) shows the correlation between *D*_KL_ values and average human scores for each of the five cohorts. Human-human correlation (right) indicates the correlation between the scores of each of 4 individual humans and the average of the remaining human scores for each cohort. Medians are indicated as gray bars. (D) Summary of song *D*_KL_ scores for the five cohorts (gray) were significantly lower than scores from a cohort of unrelated birds (blue, p < 0.01, Wilcoxon rank test) and from a cohort of ‘isolate birds’ raised without a tutor (red, p < 0.01, Wilcoxon rank test). Across all panels, bird cohort identity is indicated by color.

We evaluated Song *D*_KL_ as a holistic measure of song similarity through comparison of Song *D*_KL_ to human scoring (Fig 5). We used both Song *D*_KL_ and human scoring to assess learning in five cohorts of Bengalese finches. Each cohort was tutored with a different song (cohort tutor song). Fig 5A shows an example of the cohort tutor song for one group (top) and 5 tutee songs that illustrate a broad range in the quality of copying. For each cohort, four expert human evaluators independently estimated the similarity between each tutee’s learned song and the tutor song on a scale of 0–4, with 0 being most similar.

Across all five groups, Song *D*_KL_ and human scores were well correlated. Fig 5A shows the average human and *D*_KL_ scores assigned to 5 tutee songs from one cohort. The rank ordering of similarities for these five songs relative to the tutor song was the same for Song *D*_KL_ and human scores; the learned songs are displayed from top to bottom in order of decreasing similarity to the tutor song by both measures. Fig 5B shows the correlation between Song *D*_KL_ and average human scores for 65 birds from the 5 cohorts (r = 0.72, p<0.01). When calculated for each cohort individually, the median *D*_KL_-human correlation was high (Fig 5C, human-computer correlation). We compared these *D*_KL_-human correlations to human-human correlations. For each cohort of birds, the scores of each evaluator were correlated with the average scores provided by the other evaluators (Fig 5C, human-human correlation). Further, the correlation between *D*_KL_ and human scores was comparable to the correlation between different humans’ scores. Together, these results indicate that Song *D*_KL_ provides a holistic and automated assessment of song learning that closely parallels human evaluation.

As an additional reference for ‘poor learning’, we also computed Song *D*_KL_ relative to each cohort tutor song for two additional groups of birds: ‘isolate birds’ that were raised without exposure to any tutor, and ‘unrelated birds’ that were raised with a tutor different from any of the five cohort tutors. Song *D*_KL_ indicates information from the reference song that is missing from comparison songs. Consistent with this, *D*_KL_ for isolate songs that contain atypical vocalizations was higher than that for songs from normally tutored birds (Fig 5A, example ‘isolate song’ and Fig 5D, summary comparisons, p<0.01, Wilcoxon rank test). Similarly, values of *D*_KL_ for songs from birds that copied an unrelated tutor were also generally higher than that for songs from birds that learned from the cohort tutor (Fig 5A, example ‘unrelated song’ and Fig 5D, summary comparisons; p<0.01, Wilcoxon rank test). An exception to this could occur when the cohort tutor song was learned poorly; in such cases the ‘learned song’ could resemble an isolate song and have larger *D*_KL_ than the *D*_KL_ for birds that copied an unrelated tutor song well (For example, in Fig 5A the 'learned song' in the bottom panel had a larger *D*_KL_ than for the 'unrelated bird song'). This reflects shared spectral structure between different tutors in our colony that may be absent in poorly learned songs.

### Quantification of changes in the spectral content of song due to deafening

**Fig 6:**
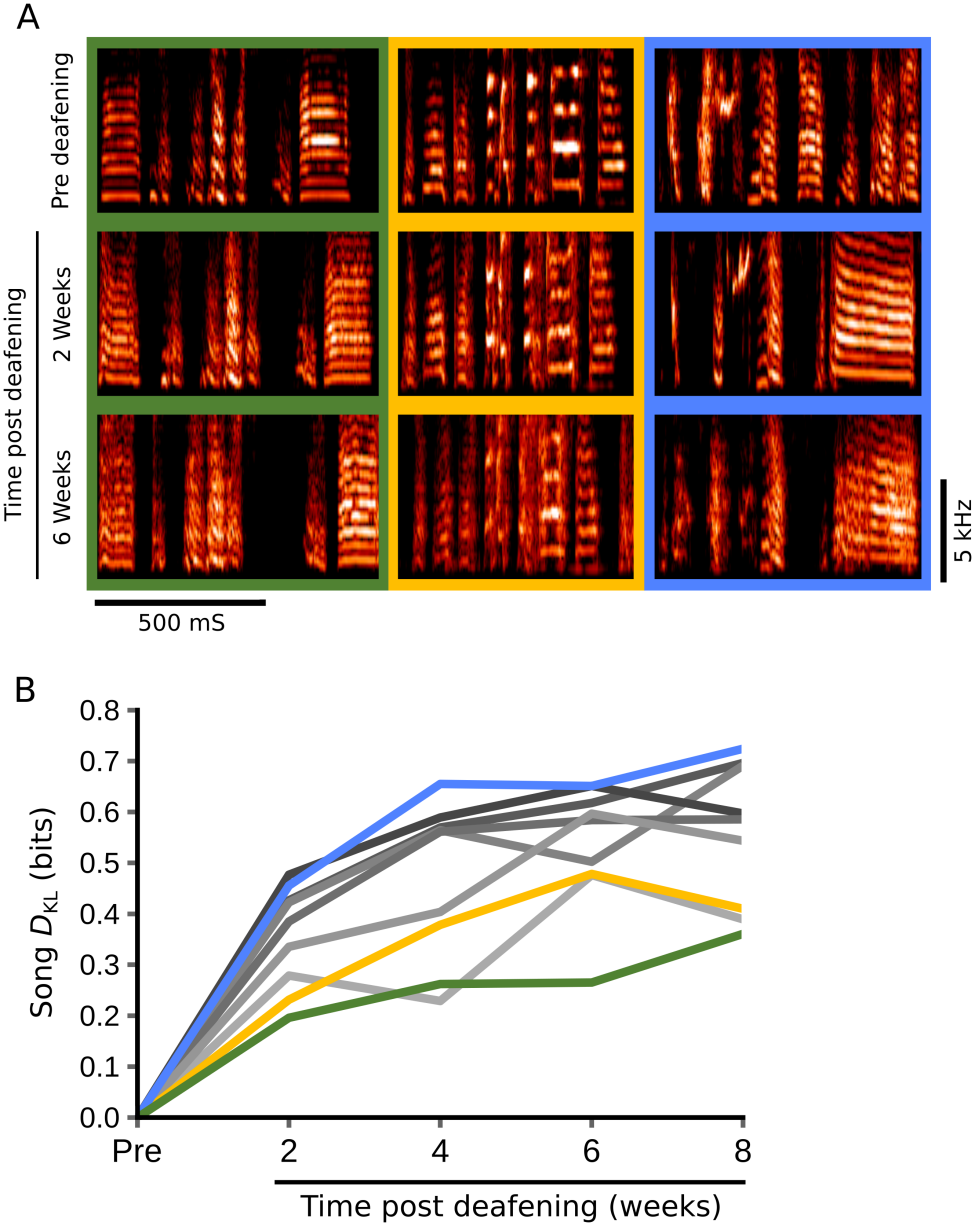
Quantification of changes to song following deafening. (A) Spectrograms from before (Pre), two weeks, and six weeks post deafening for three zebra finches demonstrate typical disruption to the spectral content of song due to deafening. (B) Song *D*_KL_ values for post deafening songs relative to baseline reference songs for nine birds at two, four, six, and eight weeks following deafening. Song *D*_KL_ values indicated by ‘Pre’ were calculated by separating the baseline reference data into two groups and comparing one group to the second group. Colors indicate bird identity, with green, yellow and blue in panels A and B illustrating data from birds that had small, intermediate and large changes to song spectral structure following deafening.

Many studies assess changes in song structure following various manipulations. One such manipulation, deafening, produces gradual deterioration of song structure and has been used extensively to provide insights into the mechanisms of song learning [6,7,14]. However, as with learning, quantitative assessment of song deterioration following deafening has often relied on human inspection [6,14]. To determine whether Song *D*_KL_ captures this deterioration we examined the songs of 9 zebra finches before and after deafening (Fig 6). We used *D*_KL_ to evaluate changes in song spectral content between baseline reference songs (before deafening) and comparison songs from the same birds two, four, six, and eight weeks post deafening. Fig 6A illustrates spectrograms from baseline songs and corresponding portions of post-deafening songs for 3 birds that qualitatively exhibited small (green), medium (yellow) and large (blue) changes to syllable spectral content. Fig 6B shows post deafening *D*_KL_ trajectories for 9 birds. *D*_KL_ calculations indicate an abrupt loss of song content within the first 4 weeks of deafening followed by a more gradual loss of content from 4–8 weeks post deafening, a trajectory consistent with song deterioration trajectories (as quantified through human inspection) reported in prior zebra finch deafening experiments [14]. These data demonstrate our approach as a sensitive method for evaluating changes in song following manipulations such as deafening.

### Robustness of similarity measures to parameter choices

Our approach is intended to provide a measure of song similarity that requires little in the way of user intervention and selection of parameters. Correspondingly, all the foregoing analyses were based on a specific instantiation using fixed values for parameters that could in principle be set by a user. Here we consider how different choices of parameter values affect similarity measures and demonstrate that *D*_KL_ measures are indeed very robust across a broad range of values. The specific parameters that we consider are 1) the number of ‘sample’ syllables drawn from both the reference song and the comparison song, 2) the number of ‘basis’ syllables used for the PSD similarity basis set, and 3) the number of mixture components in the GMM used to model the structure of each song. In addition to these numerical choices, our approach as described above uses the PSD for each syllable as a representation of the spectro-temporal complexity of song. We therefore also consider and discuss below how different choices of song representation could affect similarity measures or extend our approach to capture other aspects of song structure.

**Fig 7:**
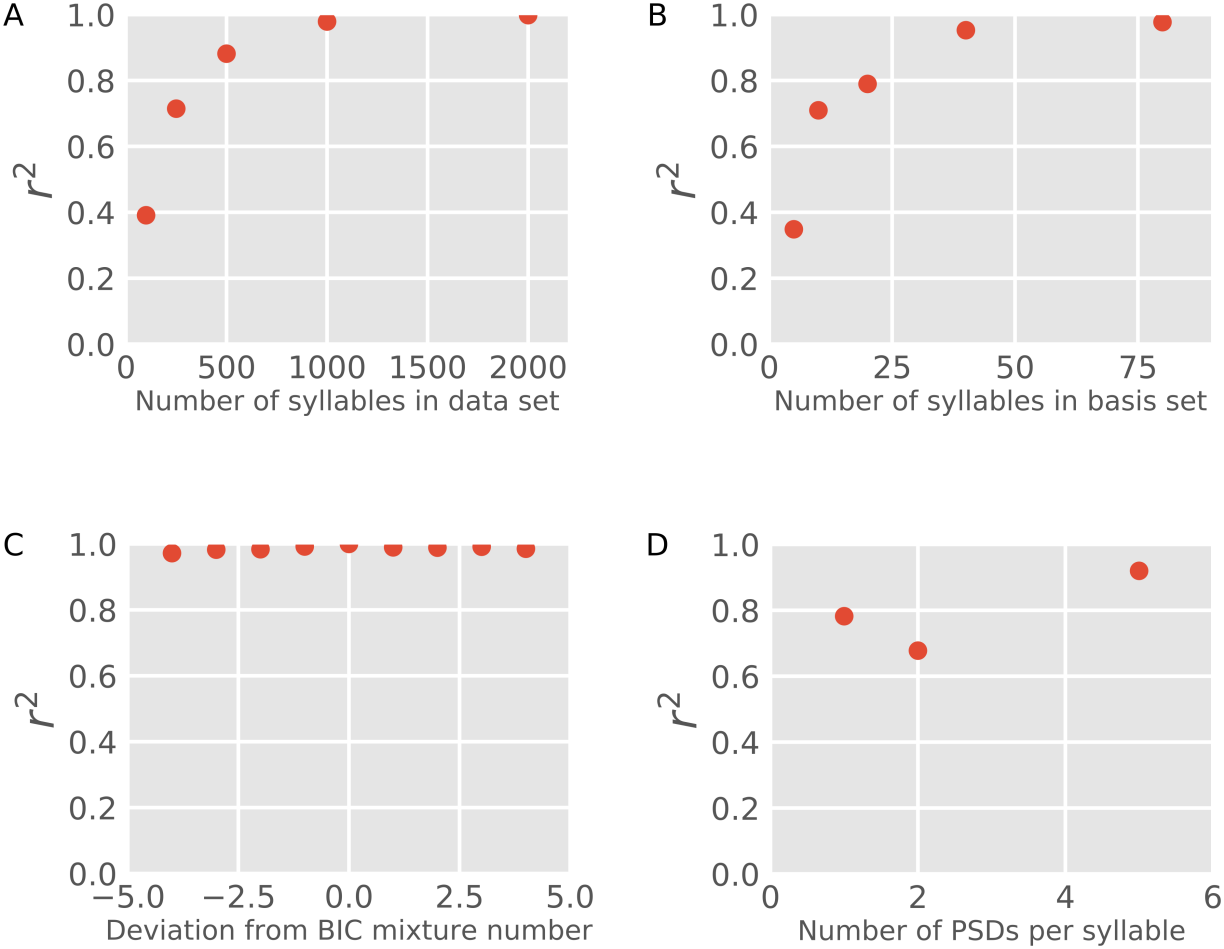
Establishment of baseline parameter values for song *D*_KL_ calculation. (A) Plot of r^2^ values for correlations between *D*_KL_ calculated using a range of input data sizes and *D*_KL_ calculated using 3000 syllables of input data. (B) Plot of r^2^ values for correlations between *D*_KL_ calculated using a range of basis set sizes and *D*_KL_ calculated using a 160 syllable basis set. (C) Plot of r^2^ values for correlations between *D*_KL_ calculated using the number of mixture components (k) determined by BIC (nBIC) and *D*_KL_ calculated using a number of mixture components ranging from nBIC-4 to nBIC+4. (D) Plot of r^2^ values for correlations between *D*_KL_ calculated using 1, 2 or 5 PSD representations of each syllable and *D*_KL_ calculated using a 10 PSD representation.

There are two numerical values important to Song *D*_KL_ that are necessarily under experimenter control: the number of sample syllables from the song to be modeled (N) and the number of syllables in the basis set (M). To determine appropriate values, we conducted numerical titrations of both the number of sample syllables in the input data set (Fig 7A and S1 Fig) and the number of syllables in the basis set (Fig 7B and S2 Fig). For each of 44 birds, Song *D*_KL_ (relative to the corresponding tutor song) was calculated using 100, 200, 500, 1000, 2000, and 3000 sample syllables from the songs that were being modeled (both reference and comparison songs). For each number of sample syllables, we computed Song *D*_KL_ values for the 44 birds and correlated these values with *D*_KL_ values computed with 3000 syllables. Not surprisingly, *D*_KL_ values with little input data showed substantial deviation from the 3000 syllable *D*_KL_ values (Fig 7A and S1A Fig). However, for sample syllable numbers above 1000, *D*_KL_-*D*_KL_ correlations approached an asymptote, indicating little change to song similarity measures above this value (Fig 7A and S1C and S1D Fig). We therefore used a fixed value of 3000 sample syllables to model song structure throughout our analysis. For this same set of 44 birds, Song *D*_KL_ values were calculated using 5, 10, 20, 40, 80, and 160 basis syllables. *D*_KL_-*D*_KL_ correlations were calculated relative to Song *D*_KL_ values derived using the 160 PSD basis set. *D*_KL_-*D*_KL_ correlations approached an asymptote for basis set numbers above 50 (Fig 7B and S2 Fig). For computational efficiency and to constrain the number of free parameters in GMMs, we therefore used a basis set of 50 syllables throughout the study. These data indicate that these *D*_KL_ based measures of song similarity are robust to changes in numbers of sample and basis syllables above threshold minimum values and therefore that our approach can be deployed effectively with fixed values of these parameters that do not require human tuning. However, this minimum threshold may scale with the complexity of the data and, for other types of data, a different minimum number of basis behaviors might be necessary.

The number of Gaussian mixtures required to model the spectral complexity of a given song was selected automatically using Bayesian Information Criteria [20]. However, the number of Gaussian mixtures can in principle be set to different values, for example in cases where the experimenter has an independent reason for modeling a song with a specific number of discrete syllable types. We therefore evaluated the robustness of Song *D*_KL_ similarity measures to variation in the number of Gaussian mixture components. Specifically, we calculated the squared error between *D*_KL_ values when calculated using the number of mixture components determined by BIC (nBIC) and *D*_KL_ values calculated using a series of other models in which the number of mixture components ranged from nBIC-4 through nBIC+4 (Fig 7C and S3 Fig). These values were used in both the reference model and the comparison model. Song *D*_KL_ was very robust to variation in the number of mixture components; squared errors for all *D*_KL_-*D*_KL_ comparisons were above 0.96 (Fig 7C, n = 44, p<0.001 for all correlations).

**Fig 8:**
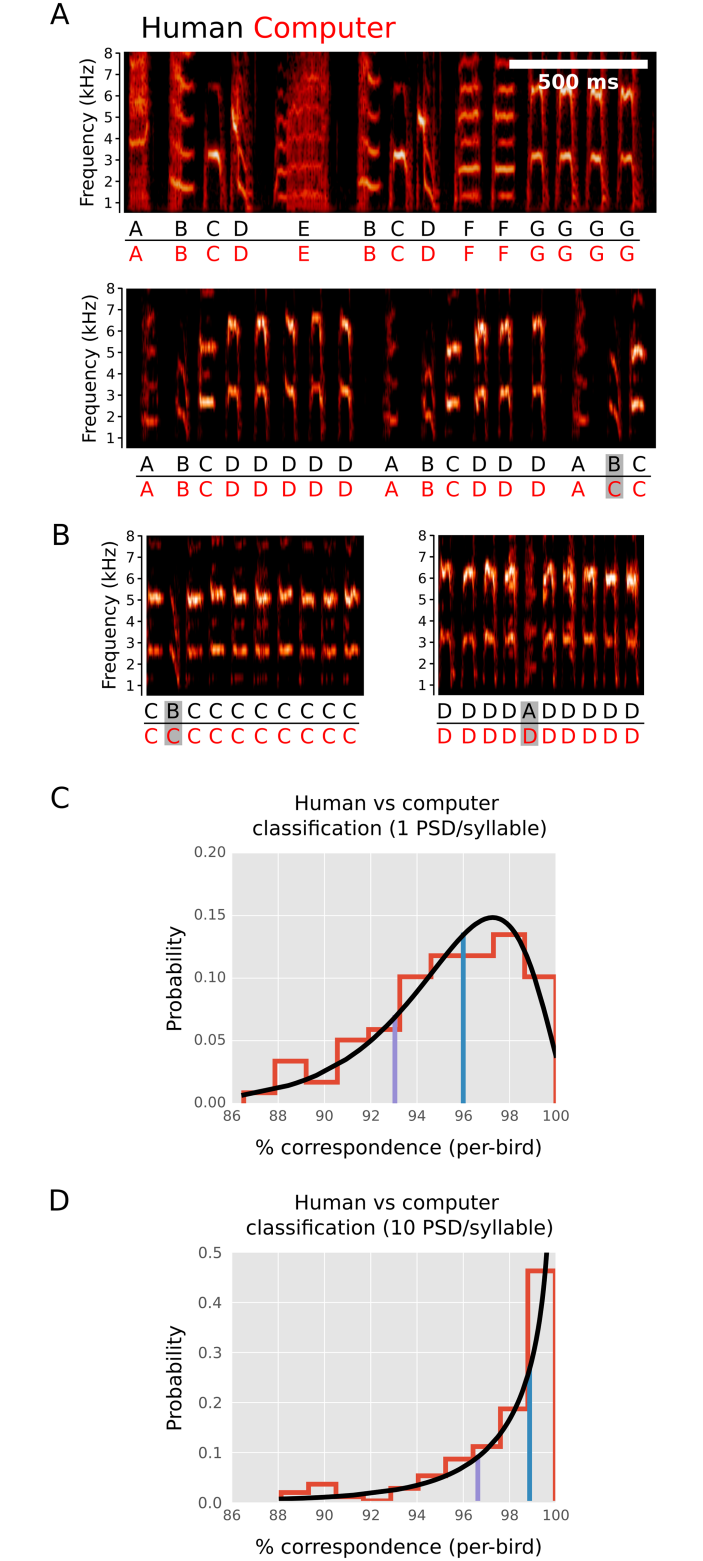
GMM derived syllable classifications are correlated with human syllable classifications. (A) Examples of labels assigned to two songs by human inspection (black) and GMM (red). For many birds, there were no differences between human assigned and GMM assigned labels (e.g. upper panel). However, for some birds, there were discrepancies (e.g. gray box, lower panel). (B) Erroneous GMM classifications can be identified by inspection of spectrograms for groups of syllables assigned to a given Gaussian mixture. Illustrated here are two examples of groups of syllables assigned to individual Gaussian mixtures where it is apparent in each case that a single syllable (gray boxes) is miss-classified relative to human assignment. For 90 animals, the number of miss-classified syllables was determined by such human inspection of groups of syllables that were assigned to each Gaussian mixture. (C) Distribution of the percent of correctly classified syllables (per-bird) is shown in red with a gamma distribution fit to these data shown in black. 50% of animals had greater than 96% correctly classified syllables (blue line) while 80% had more than 93% correctly classified syllables (purple line). (D) Distribution of the percent of correctly classified syllables per bird is shown as in C, but here with categorization carried out in which the input representation of each syllable to the GMM includes 10 PSDs evenly spaced over the duration of the syllable, rather than a single PSD for the entire syllable. Using this richer representation of a syllable, 50% of animals had more than 99% correctly classified syllables (blue line) while 80% had more than 96% correctly classified syllables (purple line).

Throughout the analyses described above, we used a single PSD to capture the spectro-temporal content of a given syllable. At each frequency represented, this single PSD encodes acoustic power averaged across the duration of the entire syllable (Fig 2B) effectively capturing syllable spectral content collapsed across time. Accordingly, time dependent spectral content is not captured by these representations and this missing content may influence Song *D*_KL_ values. We therefore compared Song *D*_KL_ calculated using single PSDs per syllable with a series of *D*_KL_ values calculated using multiple PSDs per syllable where each syllable was divided into equal duration blocks (2, 5, or 10 blocks per syllable) and PSDs were calculated for each block (Fig 7D and S4 Fig). Song *D*_KL_ calculated with single PSD syllable representations was well correlated with *D*_KL_ calculated with 10 PSDs per syllable (Fig 7D and S4A Fig; n = 44, r^2^ = 0.78). This correlation suggests that much of the spectro-temporal information in a syllable is captured in single PSD representations. However, the correlation is not perfect, and increasing the number of PSDs per syllable likely captures more spectro-temporal content. Indeed, as is discussed in the next section, increasing the number of PSDs per syllable increases the accuracy of syllable assignments (Fig 8). For the purposes of this paper, we were particularly interested in validating Song *D*_KL_ as a metric for spectral content independent of temporal information. We therefore used a single PSD to represent syllable spectral content for song modeling and calculation of *D*_KL_.

### Syllable identity assignments provided by GMMs are well correlated with human assignments

Our Song *D*_KL_ measure is intended primarily to provide a holistic measure of song similarity between a reference song and comparison song. As part of this process we model the structure of each song using a GMM in which an intuition is that each fit Gaussian corresponds approximately to what a human observer would label as a single categorically defined syllable type. The assignment of syllables to specific Gaussian mixtures is not required for the computation of Song *D*_KL_, which relies solely on the models fit to the distribution of (unlabeled) syllable similarity data. However, it is of interest to know how effectively assignment of syllables to different Gaussian mixtures results in a categorization of syllables by type that matches human labeling of syllables. Such automatic labeling of syllables has potential utility in objectively determining the number of distinct syllable types in a bird’s song repertoire and facilitating the assignment of labels corresponding to these types to large amounts of song data.

Here, we explicitly examine the correspondence between syllable labels assigned using the GMM for each song and labels assigned by expert human evaluators. For each of 90 birds, syllables were labeled by determining the maximum posterior probability for assignment of syllable identity under the GMM fitted to songs of that bird (see Methods). These classifications were then compared with classifications provided by human inspection. Fig 8A illustrates labeled syllable categories for songs from two example birds. For some birds (e.g. upper panel) there was perfect concordance between human assigned (black) and GMM assigned (red) labels (categories). However, for most birds there were some discrepancies between human and GMM assigned labels (e.g. lower panel, gray box). To determine the accuracy of GMM based classifications, for 90 birds, all assignments were inspected by an expert human observer and the classification of each was determined to be accurate or inaccurate (Fig 8B). Overall, 50% of birds had 96% or better correspondence between human and GMM assignments, while 80% of birds had 93% or better correspondence (Fig 8C). This correlation between human and GMM based syllable classification indicates that much of the complex information subjectively used by humans to classify song syllables is incorporated into the GMM models that were used to model song structure.

In this analysis, the spectral structure of each syllable was modeled using only a single PSD computed from the entire syllable (Fig 2B). To ask whether a richer spectro-temporal representation of each syllable would increase concordance between human and GMM assignments, we built GMM models as above, but with 10 separate PSDs, evenly spaced over the duration of each syllable, used as an input representation for each syllable. We previously found that this richer syllable representation had little effect on *D*_KL_ based measures of song similarity (Fig 7D). In contrast, for the specific assignment of syllable labels, this richer representation resulted in significantly improved correspondence between human assignments and GMM assignments of syllable labels (Fig 8D). With this 10 PSD representation, 50% of birds had more than 99% correctly classified syllables and 80% of birds had more than 96% correctly classified syllables. We expect that supervised labeling schemes (in which training sets of human-labeled syllables are provided) are likely to enable the closest match between automatically generated and human annotated syllables labels (e.g. [22]). However, the high concordance between categories generated by our unsupervised approach and human annotation indicates the potential utility of our method in determining syllable identities and repertoire size in an unbiased and automated fashion.

### Evaluation of *D*_KL_ in contexts beyond birdsong

We developed and tested our measure of Song *D*_KL_ to quantify the similarity between pairs of songs. However, this formulation of *D*_KL_ could in principle be applicable to quantifying similarity for other complex behaviors. Our method relies on the segmentation of song into a set of discrete and repeated motor actions (syllables), and the transformation of those syllables into similarity-space. Many other behaviors, such as human speech, handwriting, and typing also can be segmented into discrete motor actions [23–25], and hence are amenable to analysis with our approach. Below, we illustrate how human speech data can be transformed into similarity-space, and the potential utility of *D*_KL_ in characterizing similarity between sets of speech data (see also S5 Fig for examples from handwriting).

**Fig 9:**
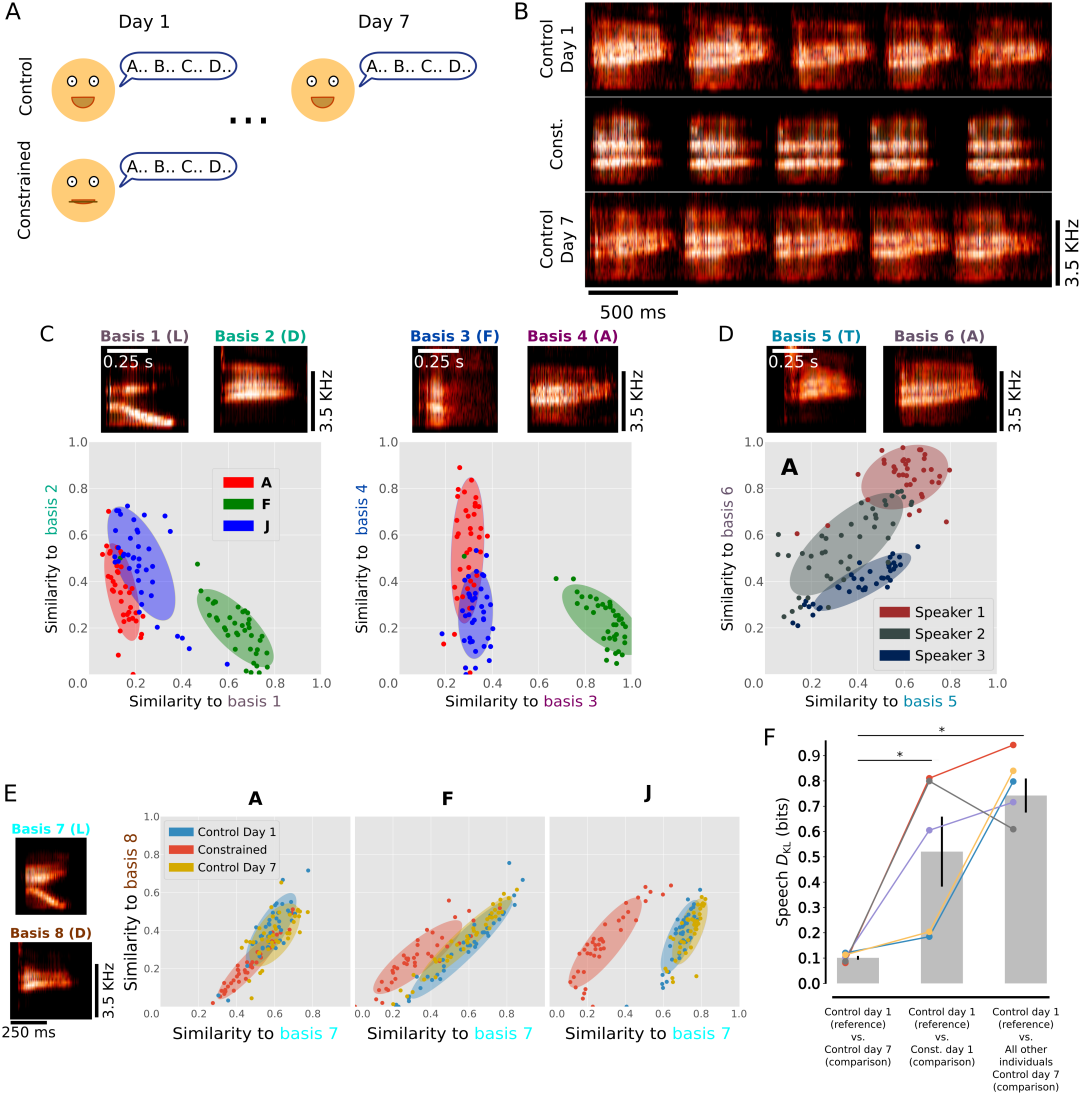
Quantification of differences between human vocalizations. (A) Schematic of experimental design. Subjects spoke the alphabet 40 times both without (Control, Day 1) and with (Constrained, Day 1) a constraint on jaw movement. Seven days after the initial recording subjects again spoke the alphabet 40 times (Control, Day 7). (B) Spectrograms of example 'A' vocalizations. Examples are drawn from data collected on day one under control conditions (top panel), day one under constraint (middle panel), and day 7 under control conditions (bottom panel). (C) Distributions of 'A' (red), 'F' (green), and 'J' (blue) vocalizations from a single participant plotted in similarity-space. In each pair of dimensions, renditions of 'A', 'F', and 'J' are well separated, though renditions of 'A' are closer to renditions of 'J' than to 'F'. Basis vocalizations are shown above each panel. (D) Distributions of 'A' vocalizations from each of three subjects plotted in similarity-space. Consistent with inter-individual differences in vocalizations, renditions of 'A' from each speaker are well separated. Basis vocalizations are shown above. (E) Distributions of 'A', 'F', and 'J' vocalizations from one subject in similarity-space. For each vocalized letter, the distribution of control-day-1 vocalizations (blue) more extensively overlap with the distribution of control-day-7 vocalizations (yellow) than with the distribution of constrained vocalizations (red). The basis vocalizations are shown at left. (F) Song *D*_KL_ values for all individuals (denoted by data color) captured differences in spectral content between control-day-1 and control-day-7 vocalizations (left column), control-day-1 and constrained vocalizations (middle column), and control-day-1 and control-day-7 vocalizations from the other subjects (right column, median value plotted for other subjects). For each data set, gray bars indicated means and black bars indicate standard errors. * = p < 0.01. For panels C-E, ellipses represent 80% confidence intervals (1.28 standard error) on Gaussian distributions fit to each set of vocalization similarities.

To evaluate the utility of our method for human speech, we quantified both naturally occurring inter-individual differences in speech sounds and experimentally driven distortions of speech sounds. Data were collected from 5 human subjects over two recording sessions (Schematized, Fig 9A). During the first session, each subject was asked to speak the alphabet 40 times in a natural fashion in order to collect a baseline set of speech sounds (‘control day 1’). Subjects were then asked to repeat the alphabet 40 times with a bite bar held between their teeth in order to constrain jaw movements and generate a distorted set of speech sounds (‘constrained’, see Methods). During the second recording session, 7 days later, subjects were again asked to speak the alphabet 40 times in a natural fashion (‘control day 7’). The speech sounds of each subject were segmented into discrete vocalizations (corresponding to the spoken letters) using amplitude thresholding. Examples of 'A' from a single subject under each condition are presented as spectrograms in Fig 9B.

We transformed speech data from each of the subjects into similarity-space, just as we had for birdsongs; each vocalization was represented as a PSD, and we measured the Euclidean distance between those PSDs and 50 basis PSDs corresponding to vocalizations drawn randomly from the entire data set. Fig 9C shows for one subject the distributions corresponding to three speech sounds (A, F and J) relative to two pairs of basis sounds. As was the case for song syllables, vocalizations corresponding to individual letters fell into distinct regions in similarity-space that could be overlapping in any given dimension, but that were generally well separated across all 50 dimensions (Fig 9C). Moreover, we found qualitatively that the distributions corresponding to a given letter differed both with speaker identity (Fig 9D) and speaking condition (Fig 9E, ‘control’ versus ‘constrained’). These data suggest that the distributions of features in similarity-space capture meaningful acoustic variation in speech sounds.

We quantified differences in sets of speech sounds by computing the speech *D*_KL_ for data sets projected into similarity-space. We first modeled the distributions of speech sounds in similarity-space using GMMs (in the identical fashion as for modeling song data). We then computed the *D*_KL_ for pairs of speech models. Consistent with the examples in Fig 9D, we found that *D*_KL_ was sensitive to differences between individuals; for each subject, the *D*_KL_ between control day 1 vocalizations for that subject and control day 7 vocalizations for the other subjects was significantly larger than the *D*_KL_ between control day 1 for that subject and control day 7 for the same subject (Fig 9F, left and right columns, p<0.01, t-test). Additionally, for each subject, the *D*_KL_ between control day 1 vocalizations and constrained vocalizations was significantly larger than the *D*_KL_ between control day 1 and control day 7 vocalizations for that subject (Fig 9F, left and middle columns, p<0.01, t-test). Together, these results indicate that *D*_KL_ calculated in this manner can quantify differences between speakers as well as changes in speech over time or across manipulations. More broadly, they suggest the potential utility of our approach in analysis of human speech and other complex behaviors that can be decomposed into sets of discrete and repeated actions.

## Discussion

We demonstrate an approach for analysis of song and song learning that is computationally efficient and automated. We use syllable spectral content to assemble statistical models for song that can then be used to estimate the amount of spectral content present in one song but absent in another. Our measure of song similarity, the Song *D*_KL_, is well correlated with holistic song similarity scores provided by expert human evaluators while providing several critical advantages. Song *D*_KL_ is automatically computed and thus consistent given the same data, where human assessment is less reliable both across individuals and over time. Because our *D*_KL_ based similarity measure is automatically and efficiently computed, we can analyze large amounts of data (many thousands of songs) facilitating dense analysis of learning across time and, for any given comparison, incorporating much more song data in assessments of learning than can be accomplished by a human evaluator. Importantly, this also obviates the need for selection of specific samples of song for comparison, and results in measurements of similarity that neither require, nor are potentially biased by, human intervention in selection of representative samples of song. Human evaluators of song learning also require species-specific training to ensure reliability and increase consistency across individuals. We show that our approach can be applied, with no modification, to two different songbird species vocalizations, indicating that this approach can be readily extended to analyze other vocalizations and other complex but similarly structured data. While no measure that only examines vocal output can indicate the degree of learning perceived by an individual bird, our approach provides an automated, holistic, and unbiased assessment of vocal learning that is well correlated with human evaluations.

Song *D*_KL_ is asymmetrical in that it estimates the amount of spectral content in a reference song that is missing from a comparison song. This asymmetry can be exploited to address distinct conceptual questions contingent on the reference-comparison relationship. In the case of birdsong, when the reference is tutor song, and the comparison is tutee song, the measure indicates how much content from the tutor song was not learned by the tutee. Reciprocally, if the reference is tutee song, and the comparison is tutor song, Song *D*_KL_ indicates how much content in the tutee song did not come from the tutor, providing an estimate of “innovation”.

We specifically focused on assessing the learning of song spectral content, but the general framework of calculating the divergence between statistical models can be extended (or restricted) to different categories of song information by changing the statistical descriptions of song. For example, the model could be extended to include, not only spectral content, but also syllable transition information using a Hidden Markov Model (HMM) with Gaussian emissions. HMMs have been effectively used in the past to model song transition structure with human assigned syllable identities [26]. If HMMs with Gaussian emissions were used in place of GMMs, the resultant statistical models would capture syllable-syllable transition probabilities in addition to the mean, distribution and covariance structure in syllable similarity-space. In this case, *D*_KL_ would indicate discrepancies not just in spectral content but also in syllable ordering. More generally, our approach to the evaluation of learning can be applied to any aspect of song that can be incorporated into a statistical model.

Beyond song learning, our approach allows fitting GMMs to sparse, high dimensional data (like PSDs of large samples of syllables). GMMs have been difficult to fit to such data partially because standard marginalization based dimensionality reduction approaches (e.g. principle components analysis) remove covariance structure that is potentially critical to fitting accurate GMMs [27]. Here we reduce the dimensionality and the sparseness of our data via calculation of syllable-syllable similarities. Our results indicate that this intuitive approach allows modeling high dimensional data sets within a framework that facilitates quantitative and meaningful comparisons. Similarity matrices are already used in the context of spectral [28] and hierarchical [29] clustering, but neither approach provides a statistical description (provided by GMMs) of data and, thus, cannot be easily used for information theoretic calculations. Our approach may have broad application to high dimensional problems where statistical descriptions can be leveraged for more accurate classification or where information theoretic calculations are desired.

## Materials and methods

### Ethics statement

All protocols were reviewed and approved by the Institutional Animal Care and Use Committee at the University of California, San Francisco. All experiments involving humans were reviewed and approved by the Institutional Review Board at the University of California, San Francisco.

### Subjects

Subjects were male Bengalese finches (*Lonchura striata domestica*), zebra finches (*Taeniopygia guttata guttata*) or human volunteers of both sexes.

### Sound recordings

For song recordings, birds were single housed in sound isolation chambers (Acoustic Systems). Songs were recorded digitally at a sampling frequency of 32 kHz and a bit depth of 16 using custom Python or LabView (National Instruments) software then stored uncompressed. Recording microphones were placed in a fixed position at the top of the cage housing the bird. For audio recordings of humans. vocalizations were recorded at 44.1 kHz at a bit depth of 16 using a field recorder (TASCAM) then stored uncompressed. Prior to further analysis, all vocalizations were digitally high-pass filtered at 500 Hz using a digitally implemented elliptical infinite impulse response filter with a passband edge frequency of 0.04 radians.

### Vocalization segmentation

Discrete units of sound separated by silence ('vocalizations') were identified based on amplitude. First an ‘amplitude envelope’ was created by rectifying the song waveform and then smoothing the waveform through convolution with an 8 ms square wave. This amplitude trace was then used, through thresholding, to identify periods of vocalization. To automatically identify a threshold capable of separating vocalizations from silence, we used Otsu's method [30]. Briefly, Otsu's method is an exhaustive search to identify a threshold that minimizes the shared variance between data above threshold and data below threshold. Once the threshold is established, 'objects' are identified as contiguous regions of the amplitude envelope over threshold. To eliminate short and spurious threshold crossing that can occur at the edge of syllables where syllable amplitude is low, any objects separated by a gap of 5 ms or less were merged, and then any objects shorter than a minimum duration, 10 ms for avian vocalizations and 50 ms for human vocalizations, were eliminated. The onsets and offsets of each 'object' were padded by 3 ms and then used to segment audio data from the original filtered waveform. These segmented vocalizations were then used in subsequent analyses.

### Power spectral density estimation

To estimate spectral content of vocalizations while removing temporal information, we calculated the power spectral density for each syllable at 2048 frequencies using Welch's method [17]. Briefly, for each syllable, successive 4096 point FFTs, each overlapping by 256 samples were calculated. Where each sample window extended beyond the syllable duration, the window was padded with zero values. These were then averaged over the duration of the syllable. As each FFT is symmetrical we used only first 2048 samples of the averaged FFT for subsequent analysis. Power in the frequency range 600 Hz– 1600 Hz (sampled at 1970 points) was used as the PSD for that vocalization.

### Similarity calculations

We transformed each PSD into a syllable similarity representation as follows. For each of M syllables, the Euclidean squared distance between the PSD of that syllable and the PSDs of a basis set of N reference syllables was calculated creating an MxN distance matrix, *D*, in which *D*_*ij*_ = ‖*p*_*i−*_*q*_*j*_‖^2^ where *p* is the vector of M syllable PSDs and *q* is the vector of N basis set syllable PSDs. The basis syllables were either drawn randomly or were the first N syllables from the set of syllables being modeled. Either method produced qualitatively similar results. We then calculated A, a similarity matrix, where *A*_*ij*_ = 1 / (*D*_*ij*_ */* max(*D*)). For every analysis presented an M of 3000 data points and N of 50 basis data points were used.

### Gaussian mixture model and parameter estimation

We modeled each set of vocalizations as a Gaussian mixture fit to the distribution of those vocalizations in similarity-space. These GMMs [18] were defined as:

$$p\left(x|\theta \right)=\overset{K}{\underset{k=\text{1}}{\sum }}\pi_{k}N(x|\mu_{k},\Sigma_{k})$$ (1)

Where *x* were the observed data after projection into similarity-space. K was the number of mixture components (analogous to syllable ‘types’), *π*_*k*_ were the mixture weights, ***μ***_*k*_ were the means, and **Σ**_*k*_ were the covariance matrices. The values of *θ* = {*π*_*k*_, *μ*_*k*_, **Σ**_*k*_} were the parameters. For parameter estimation, the values of *π*_*k*_, *μ*_*k*_, and **Σ**_*k*_ were initialized using the K-means algorithm and then estimated through standard expectation-maximization (EM). Expectation-maximization, a standard approach to parameter estimation in the context of GMMs [19], is an iterative process in which the posterior probability across all K ‘types’ is calculated for each datum in the training set given the current set of parameters (the expectation step) and these posterior probabilities are then used to update the parameter values (the maximization step). This process is repeated until the likelihood of the model converges. This procedure results in a maximum likelihood estimate for the parameters which is guaranteed to be a local maximum [19]. For this work, the implementation of expectation-maximization for GMMs in the scikit-learn software package was used [31]. Here we iterate at least 100000 times and stop at convergence. To support identification of parameters which represent a global maximum and not a local maximum, we conduct this EM procedure 5 times with different parameter initialization values. Each independent EM iteration was initialized with the output of a K-means clustering algorithm which was itself initialized with random parameter values. The best (most likely) of the 5 converged models was then selected.

### Model selection

We conducted model selection to identify the number of Gaussian mixture components needed to describe each set of vocalizations. For any given set of data, we fit a series of GMMs with increasing numbers of mixture components (*K*), ranging from 2–20 for avian vocalizations and 2–35 for human vocalizations. For each model we calculated a three-fold cross validated Bayesian Information Criterion (BIC) [20]. The BIC is a measure of model fit that is penalized for increasing model complexity. For each data set, as the number of Gaussian mixture components increased, the BIC decreased to a minimum value and then increased again as the number of mixture components exceeded the optimal value. The number of mixture components yielding the lowest BIC provided an estimate of the number of discrete vocalization 'types' present in a given data set. For each specific set of vocalization data the GMM resulting in the lowest BIC value was used in subsequent analyses.

### Estimation of song similarity

As is elaborated above, we captured vocalization spectral content as distributions in similarity-space. We quantified the difference between two songs, a reference song (i.e. a tutor song) and a comparison song (i.e. the song of a tutee) by estimating a measure of divergence between probability distributions, the Kullback-Leibler divergence (*D*_KL_) [21]. *D*_KL_ is a holistic measure that does not require selection or classification of data nor does it require direct comparisons of specific Gaussian distributions. The *D*_KL_ is defined as:

$$D_{\text{KL}}\left(P||Q\right)=H\left(P,Q\right)-H\left(P\right)$$ (2)

Where *P* and *Q* are probability distributions, *H*(*P*) is the entropy of *P* (conceptually, the amount of information required to encode data from *P*) and *H*(*P*,*Q*) is the cross-entropy between *P* and *Q* (conceptually, the amount of information required to encode data from *P* using the distribution *Q*). Hence, the difference between *H*(P) and *H*(*P*,*Q*) is the amount of information lost when using the distribution *Q* to encode data from the distribution *P*[32]. In the context of song learning, if *P* is the tutor-song distribution and *Q* is the tutee-song distribution, then *D*_KL_(*P||Q*) is the amount of information lost when using the tutee song to encode the tutor song; if the distribution of tutee vocal data is similar to the distribution of tutor vocal data (i.e. tutor song is well learned by the tutee) then *D*_KL_(*P||Q*) will be low.

In computing *D*_KL_, we have available only empirically measured samples of song data, but not the true distributions from which those data were derived. We therefore approximated the distributions of song data in functional forms, namely Gaussian mixture models (detailed above). Given two GMMs, one representing the distribution of data from a reference song (here we refer to the model for the true distribution *P* as $\hat{P}$), and a second model representing the distribution of data from a comparison song ($\hat{Q}$) we would then like to determine $D_{\text{KL}}\left(\hat{P}||\hat{Q}\right)$, the information lost when using $\hat{Q}$ (the GMM capturing the comparison song, e.g. tutee song) to represent $\hat{P}$ (the GMM capturing the reference song, e.g. tutor song). There is no closed form solution for the *D*_KL_ between two GMMs. We therefore calculated *D*_KL_ as the difference between two cross entropies which can themselves be estimated from sample data. This is a standard approach to *D*_KL_ estimation that has been shown to provide an unbiased estimate of the *D*_KL_ between two GMMs even with small sample sizes [33]. We estimated $D_{\text{KL}}\left(\hat{P}||\hat{Q}\right)$ as:

$$D_{\text{KL}}\left(\hat{P}||\hat{Q}\right)=H\left(P,\hat{Q}\right)-H\left(P,\hat{P}\right)$$ (3)

Where $H\left(P,\hat{Q}\right)$ is the cross-entropy between the GMM for the comparison song model and the true reference song distribution and $H\left(P,\hat{P}\right)$ is the cross-entropy between the GMM for the reference song model and the true reference song distribution. If $\hat{P}$ is equal to *P*, and $\hat{Q}$ is equal to *Q*, then $D_{\text{KL}}\left(\hat{P}||\hat{Q}\right)$ is simply *D*_KL_(*P||Q*). Thus, $D_{\text{KL}}\left(\hat{P}||\hat{Q}\right)$ provides an estimate of *D*_KL_(*P||Q*).

To calculate $D_{\text{KL}}\left(\hat{P}||\hat{Q}\right)$ we must calculate $H\left(P,\hat{P}\right)$ and $H\left(P,\hat{Q}\right)$. However, as the true distribution *P* is unknown, we estimated these cross-entropies using samples from *P*. We estimated $H\left(P,\hat{P}\right)$ as:

$$H\left(P,\;\hat{P}\right)=-\sum_{n=\text{1}}^{N}\frac{\text{1}}{N}p_{n}{\text{log}}_{\text{2}}\hat{p}$$ (4)

We estimate $H\left(P,\hat{Q}\right)$ as:

$$H\left(P,\;\hat{Q}\right)=-\sum_{n=\text{1}}^{N}\frac{\text{1}}{N}p_{n}{\text{log}}_{\text{2}}\hat{q}$$ (5)

Where *p* are samples from the true distribution of *P*. In this case, *p* are samples of reference song data which were not used estimate the parameters in $\hat{p}$. $\hat{p}(p)$ and $\hat{q}(p)$ are the likelihood of *p* under the models *p* and *q* respectively. This formulation is well established as an estimator of cross-entropy [34]. Substitution of Eqs 4 and 5 into Eq 6 provides our estimator for $D_{\text{KL}}\left(\hat{P}||\hat{Q}\right)$.

$$D_{\text{KL}}\left(\hat{P}||\hat{Q}\right)=-\sum_{n=\text{1}}^{N}\frac{\text{1}}{N}p_{n}{\text{log}}_{\text{2}}\hat{p}+\sum_{n=\text{1}}^{N}\frac{\text{1}}{N}p_{n}{\text{log}}_{\text{2}}\hat{q}$$ (6)

This same formulation has been shown to provide unbiased estimates of *D*_KL_ between two GMMs across a range of input samples sizes, though as sample sizes decrease, the variance of the estimate increases [33]. Here, we found empirically that estimates of *D*_KL_ were relatively constant for sample sizes above 1000 (Fig 7 and S1 Fig). For analyses presented in Results we use 3000 samples from the tutor song in estimating $D_{\text{KL}}\left(\hat{P}||\hat{Q}\right)$. when $D_{\text{KL}}\left(\hat{P}||\hat{Q}\right)$ is estimated using models derived from song we refer to this value as the Song *D*_KL_.

### Syllable classification

For each syllable, the GMM based syllable classification was calculated as the maximum posterior probability over the *K* syllable 'types' defined by each of the Gaussian mixtures in the fit model. Calculated as:

$$z_{n}=\underset{k}{argmax}\;p(k|x_{n},\theta )$$ (7)

$$=\underset{k}{argmax}\frac{\pi_{k}N(x_{n}|\mu_{k},\Sigma_{k})}{\sum_{j=\text{1}}^{k}\pi_{j}N(x_{n}|\mu_{j},\Sigma_{j})}$$ (8)

Where *z*_*n*_ is the assigned syllable type.

### Human assessment of song similarity

Song *D*_KL_ estimates of song similarity were compared with similarity estimates made by humans experienced in song analysis. We considered 5 groups of songs, corresponding to 5 nests in our colony. Each group consisted of a tutor reference song, produced by the adult male breeder in the nest, and multiple tutee comparison songs, between 8 and 20 tutee songs per group, corresponding to juveniles that were hatched and raised to independence in the nest of the adult male tutor. These groups were chosen such that each of the tutor songs was qualitatively distinct, and each group of tutees expressed a broad range in the quality of tutor song copying. For each group of songs, human judges that had extensive experience in song analysis were presented with spectrograms (frequency range from 500–10000 Hz) representing four examples of tutor song and four examples of each tutee song. Each example consisted of four seconds of each song presented on the same time scale. Human judges assigned a score between zero (high similarity to tutor), and four (low similarity to tutor), to each tutor-tutee song pair.

### Deafened birds

Deafening data were presented previously in Kojima et. al. 2013 [35]. For each of seven birds, song was recorded before deafening and at two, four, six, and eight weeks post deafening. For each bird the Song *D*_KL_ at any time post-deafening was calculated with reference to the pre-deafening song. To establish a pre-deafening baseline Song *D*_KL_, song recorded prior to deafening was divided into two groups and *D*_KL_ was calculated between these groups.

### Evaluation of human vocalizations

Vocalizations from five subjects, three male and two female, were recorded during two sessions separated by a week. In the first session subjects spoke the alphabet 40 times naturally, and, immediately afterward, spoke the alphabet 40 times while holding a flat wooden dowel in their teeth (‘bite bar’). The bite bar constrained jaw movements relative to natural speech without directly restricting movement of the other speech articulators. In the second session participants again spoke the alphabet 40 times naturally. For each session audio was recorded at 44 kHz and a bit depth of 16. These recordings were processed identically to recordings of birdsong with the following change. Following identification of continuous vocalizations above an amplitude threshold, vocalizations shorter than 50 ms were excluded from further analysis.

## Supporting information

S1 Fig Song *D*_KL_ is consistent when greater than 1000 input syllables are used. (A-D) Correlation between *D*_KL_ calculated with 3000 syllables of input data vs. 100, 500, 1000, and 2000 syllables of input data. *D*_KL_ values calculated from 44 song comparisons are plotted. Unity line is shown in red.
(TIFF)

S2 Fig Song *D*_KL_ is consistent when greater than 40 basis syllables are used. (A-E) Correlation between Song *D*_KL_ calculated for 160 basis syllables vs. 5, 10, 20, 40, and 80 basis syllables. *D*_KL_ values calculated from 44 song comparisons are plotted. Unity line is shown in red.
(TIFF)

S3 Fig Song *D*_KL_ is robust to changes in the number of Gaussian mixture components. (A-H) Correlation between *D*_KL_ calculated for models with the number of mixture components indicated by the BIC (nBIC) and models with deviations from this value ranging from nBIC-4 to nBIC+4. *D*_KL_ values calculated from 44 song comparisons are plotted. Unity line is shown in red.
(TIFF)

S4 Fig The influence of increasing temporal sampling density of syllable spectral content on Song *D*_KL_. (A-C) Correlation between *D*_KL_ derived from syllables represented with 10 PSDs, evenly distributed across syllable duration, and syllables represented with one, two and five PSDs. *D*_KL_ values calculated from 44 song comparisons are plotted. Unity line is shown in red.
(TIFF)

S5 Fig Distributions of handwritten numerals in numeral similarity-space are separated based on numeral identity and are distributed approximately normally in any given dimension. Handwritten numerals (zero and one) for this analysis were drawn from the MNIST handwritten digit data set. The data set contains examples of each numeral from many individuals. (A) Examples of zeros and ones from the dataset. (B-F) Distributions of similarities between 3000 samples each of zero (red) and one (blue) relative to basis numerals. Basis numerals for each panel are shown at bottom and left. Similarity between each pair of handwritten numerals was computed as the Euclidean squared distance between the pixel values (28x28) corresponding to each numeral, normalized to a range 0–1 (with 1 indicating greater similarity). Examination of the marginal distributions (right and top) for each panel suggest that Gaussian modeling would capture much of sample distribution (in similarity-space) for these numerals. In several of the dimensions depicted the distributions corresponding to samples of ‘zero’ and the distributions corresponding to samples of 'one' are well separated (e.g. panel B and C). When each handwritten numeral was represented by its similarities to 50 basis numerals our method provided a classification for each rendition of zero and one that corresponded with the human classification in 98.2% of all cases (n = 6000). These data suggest that transformation of handwriting samples into similarity-space structures data in a manner that may be amenable to analysis through the same type of statistical modeling that we have demonstrated for song analysis. For panel B-F ellipses are 80% confidence intervals (1.28 standard error) derived from a multivariate Gaussian fit to each set of numeral similarities.
(TIFF)

S1 Script This script implements the Song *D*_KL_ calculations detailed in the paper. It calculates the Song *D*_KL_ between two sets of song data. Details and requirements for usage are in the script.
(PY) Click here for additional data file.

S2 Script This script implements the Gaussian mixture model selection process detailed in the paper. It provides an estimate of the number of syllables present in a specific set of songs. It is intended to be used to estimate the number of syllables present in a given set of song data prior to calculating the Song *D*_KL_. Details and requirements for usage are provided in the script.
(PY) Click here for additional data file.
