# Supplementary figures and images for: An automated approach to the quantitation of vocalizations and vocal learning in the songbird
Source: PLoS Comput Biol. 2018 Aug 31;14(8):e1006437. doi: 10.1371/journal.pcbi.1006437 (PMC6136806; doi:10.1371/journal.pcbi.1006437)

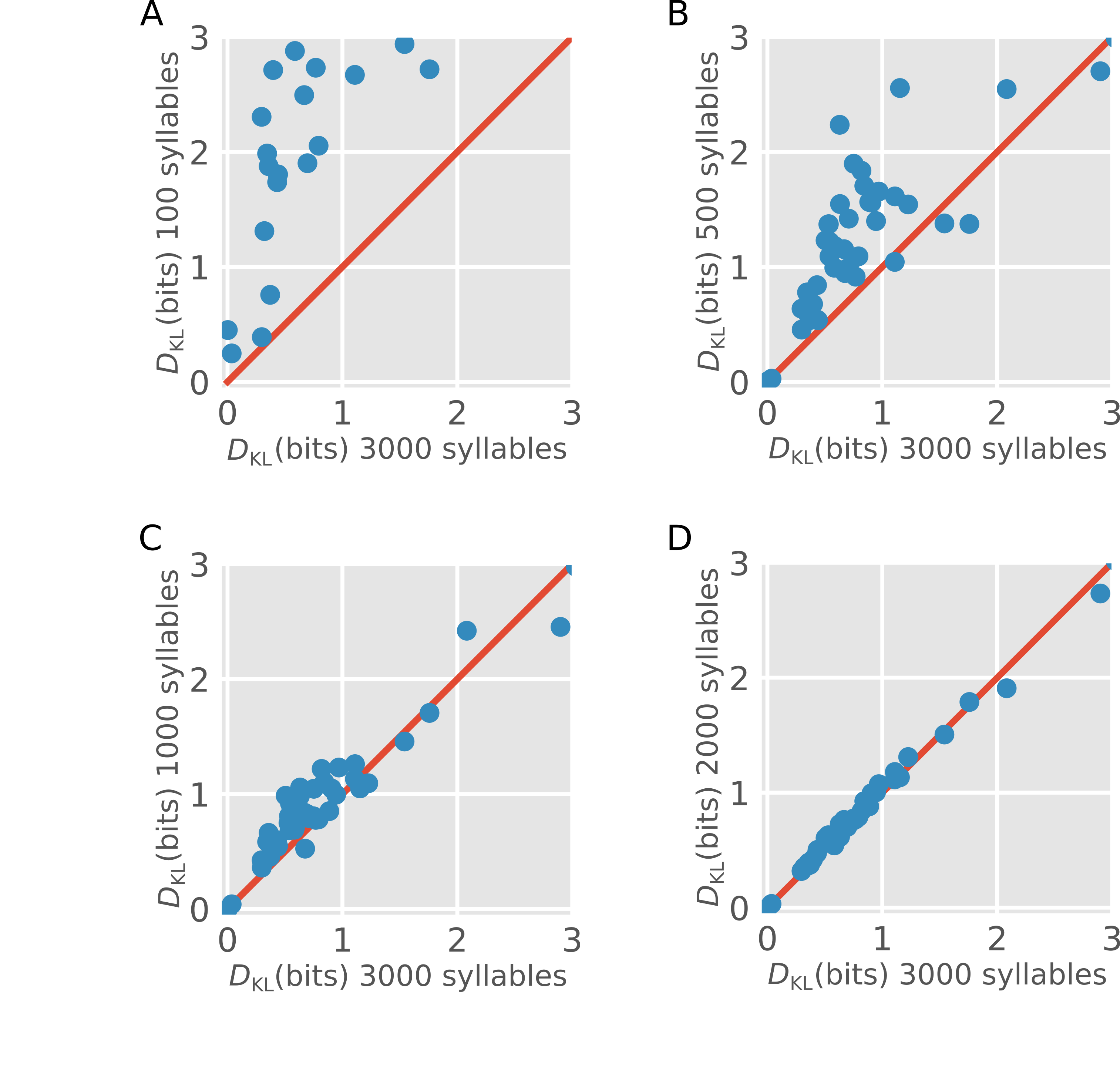

Supplement: S1 Fig — (A-D) Correlation between DKL calculated with 3000 syllables of input data vs. 100, 500, 1000, and 2000 syllables of input data. DKL values calculated from 44 song comparisons are plotted. Unity line is shown in red. (TIFF) [file pcbi.1006437.s001.tiff]

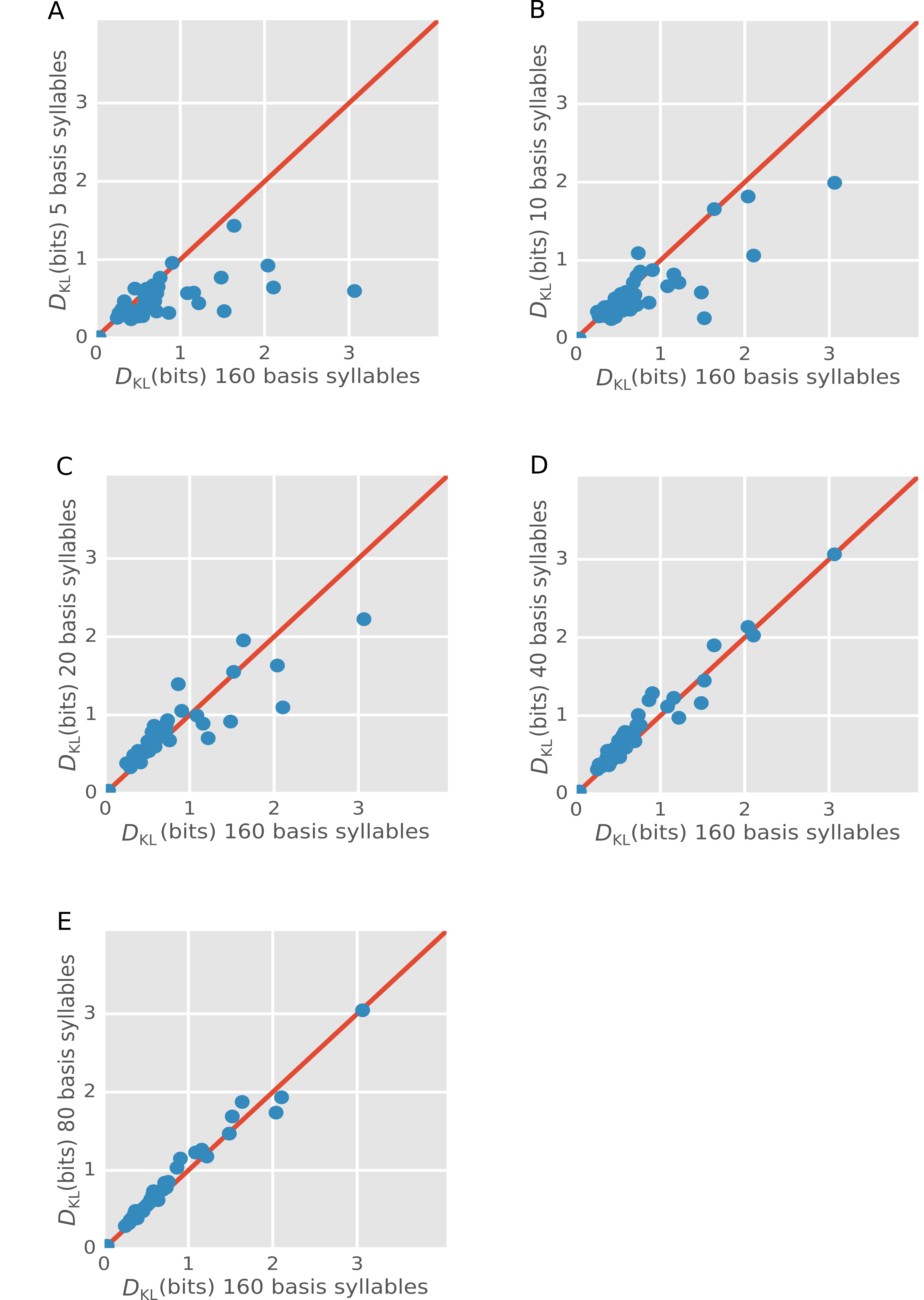

Supplement: S2 Fig — (A-E) Correlation between Song DKL calculated for 160 basis syllables vs. 5, 10, 20, 40, and 80 basis syllables. DKL values calculated from 44 song comparisons are plotted. Unity line is shown in red. (TIFF) [file pcbi.1006437.s002.tiff]

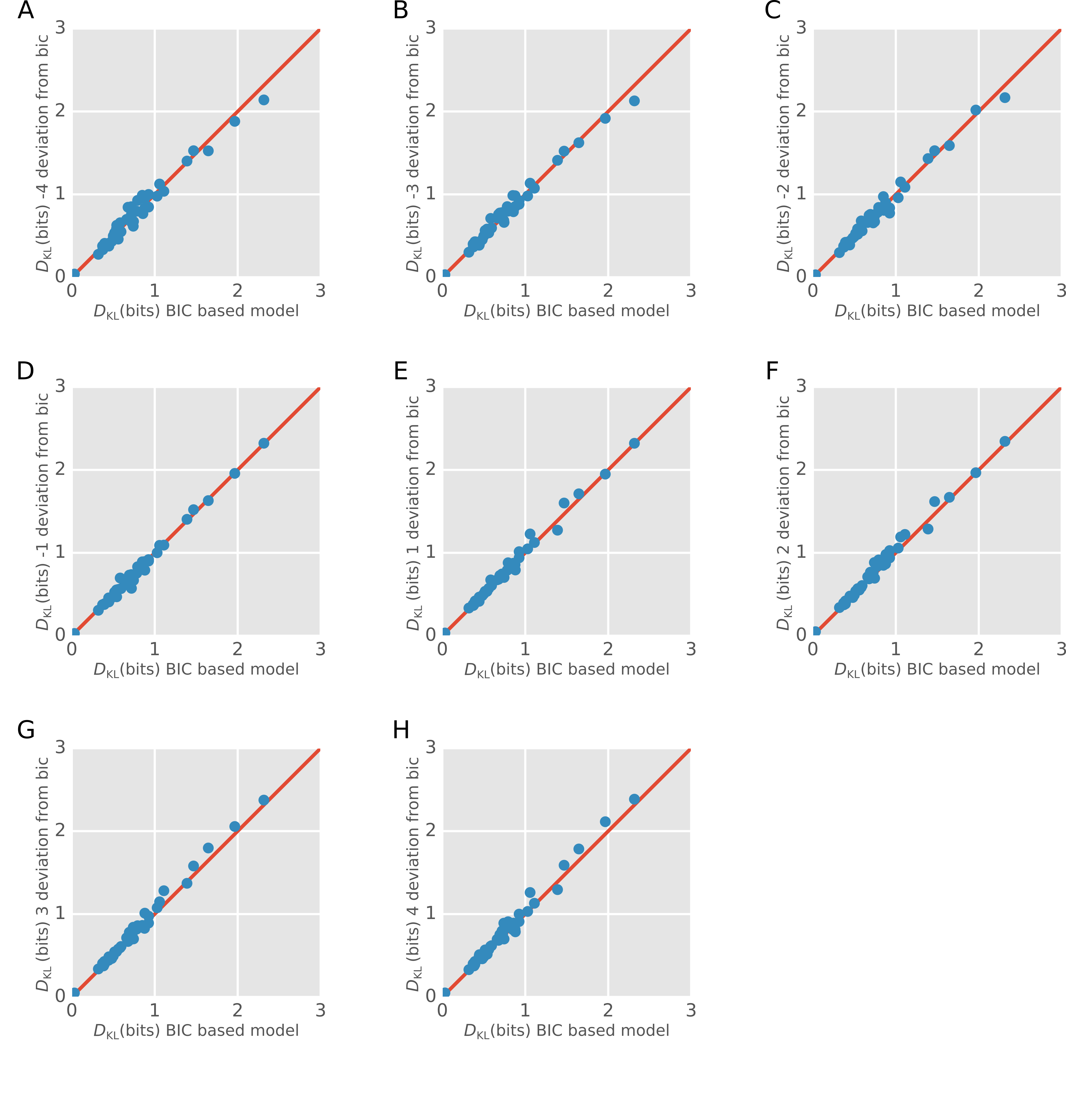

Supplement: S3 Fig — (A-H) Correlation between DKL calculated for models with the number of mixture components indicated by the BIC (nBIC) and models with deviations from this value ranging from nBIC-4 to nBIC+4. DKL values calculated from 44 song comparisons are plotted. Unity line is shown in red. (TIFF) [file pcbi.1006437.s003.tiff]

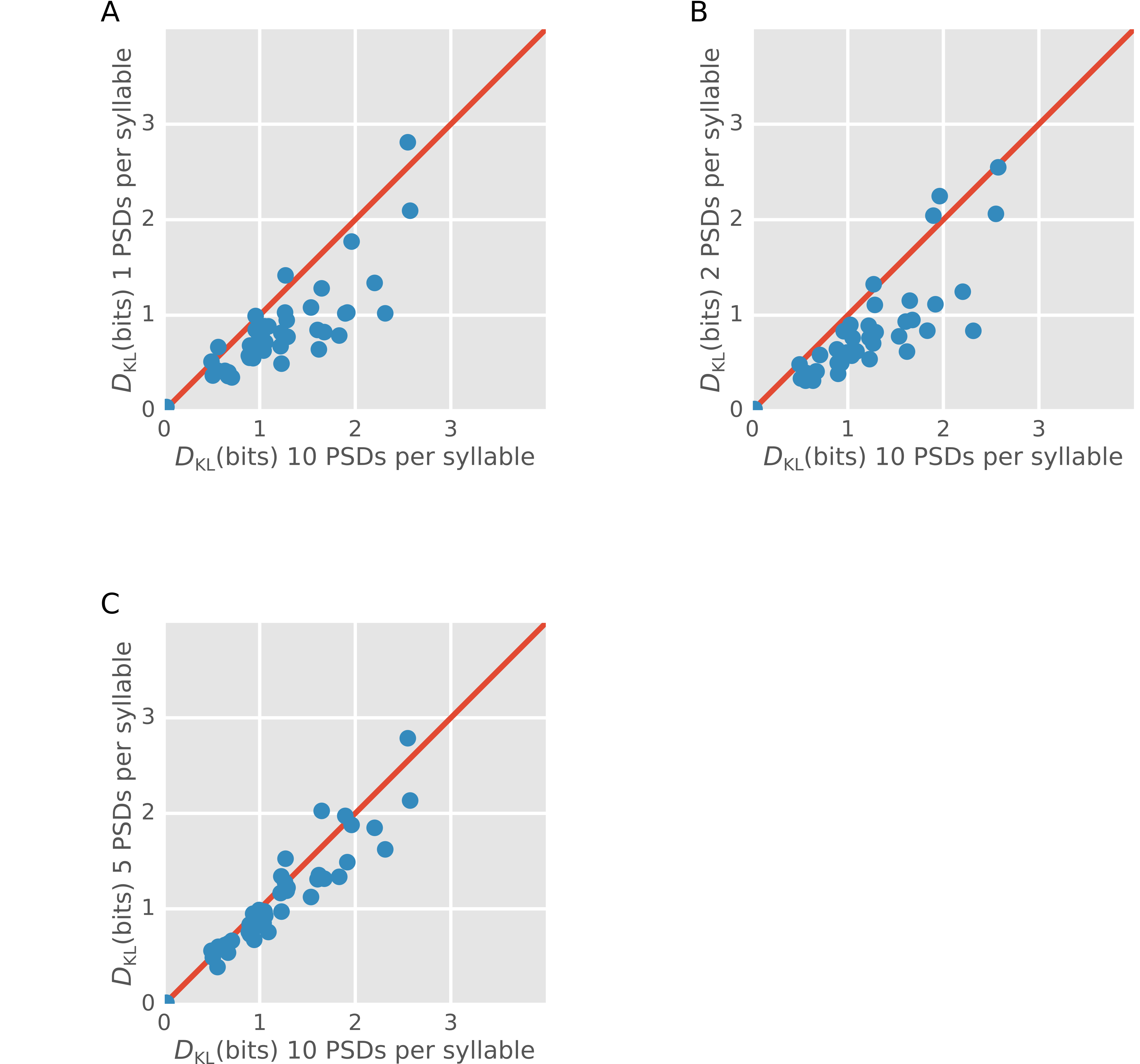

Supplement: S4 Fig — (A-C) Correlation between DKL derived from syllables represented with 10 PSDs, evenly distributed across syllable duration, and syllables represented with one, two and five PSDs. DKL values calculated from 44 song comparisons are plotted. Unity line is shown in red. (TIFF) [file pcbi.1006437.s004.tiff]

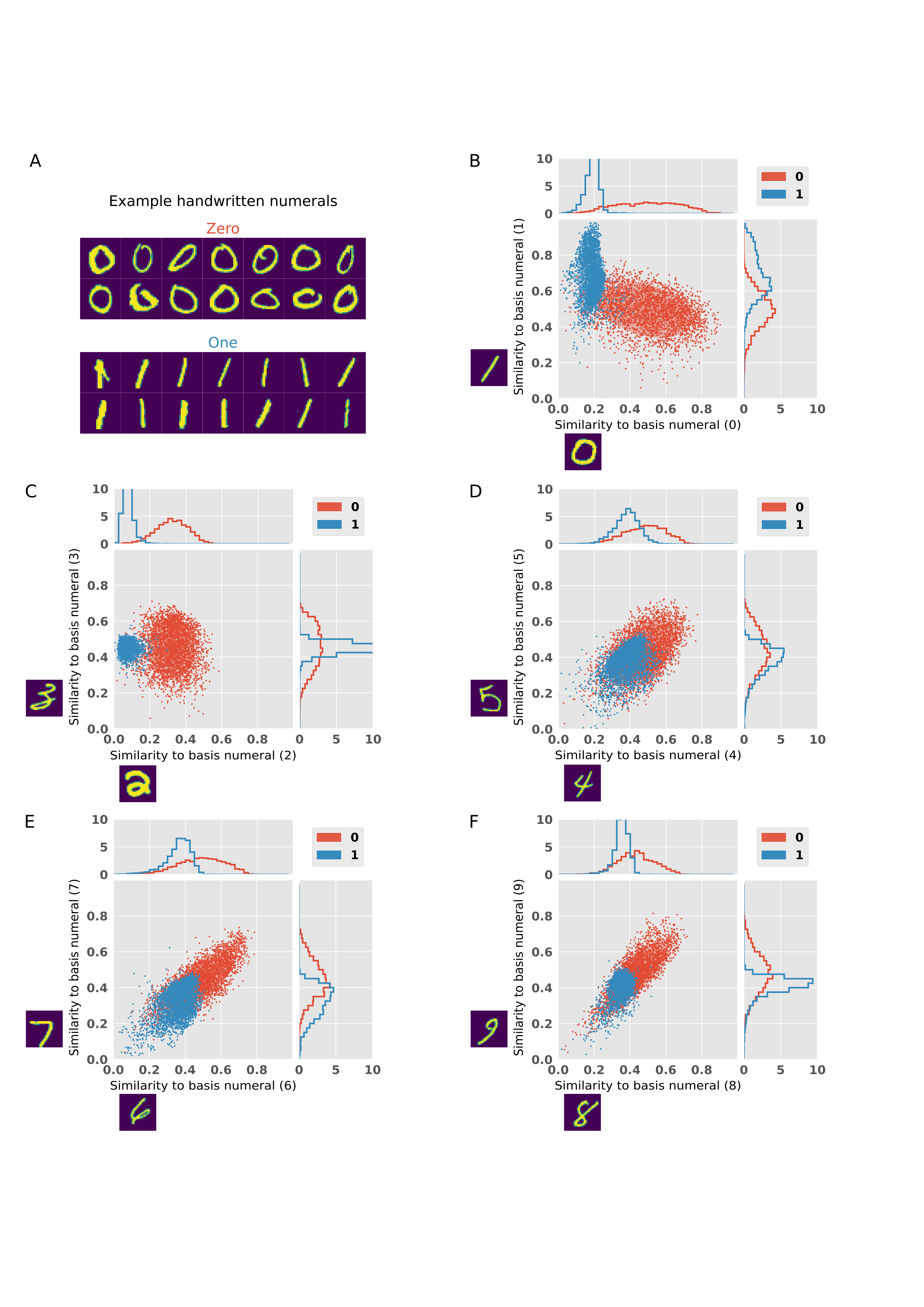

Supplement: S5 Fig — Handwritten numerals (zero and one) for this analysis were drawn from the MNIST handwritten digit data set. The data set contains examples of each numeral from many individuals. (A) Examples of zeros and ones from the dataset. (B-F) Distributions of similarities between 3000 samples each of zero (red) and one (blue) relative to basis numerals. Basis numerals for each panel are shown at bottom and left. Similarity between each pair of handwritten numerals was computed as the Euclidean squared distance between the pixel values (28x28) corresponding to each numeral, normalized to a range 0–1 (with 1 indicating greater similarity). Examination of the marginal distributions (right and top) for each panel suggest that Gaussian modeling would capture much of sample distribution (in similarity-space) for these numerals. In several of the dimensions depicted the distributions corresponding to samples of ‘zero’ and the distributions corresponding to samples of 'one' are well separated (e.g. panel B and C). When each handwritten numeral was represented by its similarities to 50 basis numerals our method provided a classification for each rendition of zero and one that corresponded with the human classification in 98.2% of all cases (n = 6000). These data suggest that transformation of handwriting samples into similarity-space structures data in a manner that may be amenable to analysis through the same type of statistical modeling that we have demonstrated for song analysis. For panel B-F ellipses are 80% confidence intervals (1.28 standard error) derived from a multivariate Gaussian fit to each set of numeral similarities. (TIFF) [file pcbi.1006437.s005.tiff]
